# Alcohol Withdrawal and the Associated Mood Disorders—A Review

**DOI:** 10.3390/ijms232314912

**Published:** 2022-11-29

**Authors:** Helena Hui Lin Ngui, Audrey Siew Foong Kow, Sally Lai, Chau Ling Tham, Yu-Cheng Ho, Ming Tatt Lee

**Affiliations:** 1 Faculty of Pharmaceutical Sciences, UCSI University, Kuala Lumpur 56000, Malaysia; 2 Department of Biomedical Sciences, Faculty of Medicine and Health Sciences, Universiti Putra Malaysia, Serdang 43400, Malaysia; 3 School of Medicine, College of Medicine, I-Shou University, Kaohsiung City 82445, Taiwan

**Keywords:** alcohol, substance abuse, anxiety, depression, oxidative stress, withdrawal

## Abstract

Recreational use of alcohol is a social norm in many communities worldwide. Alcohol use in moderation brings pleasure and may protect the cardiovascular system. However, excessive alcohol consumption or alcohol abuse are detrimental to one’s health. Three million deaths due to excessive alcohol consumption were reported by the World Health Organization. Emerging evidence also revealed the danger of moderate consumption, which includes the increased risk to cancer. Alcohol abuse and periods of withdrawal have been linked to depression and anxiety. Here, we present the effects of alcohol consumption (acute and chronic) on important brain structures—the frontal lobe, the temporal lobe, the limbic system, and the cerebellum. Apart from this, we also present the link between alcohol abuse and withdrawal and mood disorders in this review, thus drawing a link to oxidative stress. In addition, we also discuss the positive impacts of some pharmacotherapies used. Due to the ever-rising demands of life, the cycle between alcohol abuse, withdrawal, and mood disorders may be a never-ending cycle of destruction. Hence, through this review, we hope that we can emphasise the importance and urgency of managing this issue with the appropriate approaches.

## 1. Introduction

Ethanol, also known as ethyl alcohol, is the second member of a family of organic chemical compounds known as alcohol. Every member of this homologous series has at least one hydroxyl group (-OH) in its molecule [1]. It is a psychoactive ingredient of a wide range of alcoholic beverages such as beer, wine, cider, malt liquor, and distilled spirits with dependance producing properties. Recreational alcohol consumption has been incorporated into daily socializing activities of many societies [2]. Unlike marijuana and other psychoactive drugs, alcohol consumption is legally and socially acceptable in most nations [2]. However, alcohol is fully banned in Middle Eastern countries such as Saudi Arabia, Iran, and Kuwait. However, in Sudan, it is only banned among Muslims [3]. Besides this, regulations on alcohol sales and use can be seen in many nations, as well as amendments and the enforcement of strict laws against underage drinking and drunk driving.

According to the World Health Organization, three million deaths, as well as poor health reported from various countries across the globe, emerged because of alcohol consumption. In the United States, excessive use of alcohol is the fifth leading risk factor for premature death and disability. A large of this is a result of high rates of alcohol use disorder. As defined in the Diagnostic and Statistical Manual for Mental Disorders, 5th edition, alcohol use disorder is a pattern of alcohol consumption that leads to problems associated with two or more potential symptoms of alcohol use disorder. These include tolerance, withdrawal, inability to control drinking, neglect of activities, craving, excessive consumption of alcohol or over a long period, inability to fulfill major role obligations, and so on [4]. Individuals from disadvantaged socioeconomic hierarchies are especially vulnerable to alcohol-related health issues [2].

Reported cases of domestic violence, particularly sexual violence directed towards women, are found to be one of the outcomes of dangerous alcohol consumption. Such abusive behaviors have inevitably impacted women mentally, physically, sexually, as well as exposing them to a higher risk of HIV infection [5]. Apart from that, there are many alcohol-related emergencies that arise due to unmoderated alcohol consumption. According to the International Classification of Diseases, alcohol-related emergencies are categorized as psychiatric, gastrointestinal, intoxication-related, and more. In the most recent COVID-19 pandemic, a comparison study showed that there was a 13.1% increase in alcohol-related emergency visits in Ontario, Canada itself [6]. The ICD-11 categorization is inclusive of alcohol-induced delirium, unspecified psychotic disorders, duodenitis, polyneuropathy, gastric ulcers, and much more [7]. A surge in complicated alcohol withdrawal, including suicides, methanol toxicity due to consumption of methanol or household products, blindness, putamen necrosis, subcortical white matter hemorrhage, and deaths, was reported during the COVID-19 lockdown period around the world. A post-lockdown report from Italy also showed significant increase in alcohol intoxication cases in hospital emergency departments compared to before the lockdown [8].

Although the consumption of low to moderate doses of alcohol is deemed a tonic for the cardiovascular system and has potential protective effects against type II diabetes [9], excessive consumption, such as binge drinking at a party, is detrimental to health [4]. It may lead to brain damage [10], alcoholic liver disease [11], and cancers [12]. Although these detrimental health effects are mainly due to excessive consumption, we should not disregard the health impact of the moderate use of alcohol. For instance, data collected from the Million Women Study in the United Kingdom, involving 1,280,296 middle-aged women, showed that low to moderate alcohol consumption increases the incidences of certain cancers. The risk of breast cancer increases by 11, cancers of the oral cavity and the pharynx increase by 1, rectum cancer increases by 1, and cancers of the esophagus, larynx, and liver increase by 0.7 per 1000 women [13]. There was a minimal increased risk of overall cancer in men and women implicated from low to moderate drinking, concluded Cao et al. in 2015. Light to moderate drinkers’ relative risks of total cancer was 1.02 in women and 1.03 in men compared to non-drinkers of respective genders [14]. The impairment of rational and moral judgement under the influence of alcohol also increases the risk of contracting sexually transmitted diseases such as HIV infection, syphilis, and so on because of unprotected sex and/or sex with multiple partners [15]. In addition, frequent consumption in large amounts is found to be associated with increased risk of breast cancer in females in a dose-response manner among smokers and non-smokers [16]. Moreover, alcohol consumption during pregnancy can result in foetal alcohol spectrum disorder, consisting of a vast range of clinical presentations. Such a disorder is usually manifested as intrauterine growth retardation (microcephaly, low birth weight), abnormal facial features (low nasal bridge, smooth philtrum and micrognathia), and mental retardation [17]. There is no known safe amount of alcohol use during pregnancy, regardless of the type of alcohol. Hence, abstinence from alcohol is strongly recommended for pregnant women.

## 2. Pharmacodynamics—The Target of Ethanol

According to a World Health Organization statistical report, the global burden of ethanol abuse among male and female populations is 7.1% and 2.2%, respectively. However, its destructive effect on human health and society is often underestimated [2]. It is important to note that the elucidation of the exact mechanism of action of ethanol, as well as its molecular targets, remain as a study subject among researchers. Nevertheless, several theories have been established with respect to the consideration of several ion channels, such as GABA_A_ [18,19,20], N-methyl-D-aspartate (NMDA), glycine receptors, as well as the alcohol dehydrogenase enzyme [19].

### 2.1. Ethanol and GABA_A_ Receptors

Although several types of ion channels are found to be involved in ethanol-mediated central nervous system disorders, a wealth of evidence has accumulated over the years suggesting that the primary mechanism of action of ethanol responsible for both short and long-term effects is GABA_A_ receptor positive allosteric modulation [18,20,21]. The GABA_A_ receptor is a ligand-gated ion channel, with GABA being the endogenous agonist [22]. For most GABA_A_ receptors in the central nervous system, the central ion channel is surrounded by two α1 subunits, two β2 subunits, and one γ2s subunit, which collectively form a pentamer [18,21]. The composition of subunits of GABA_A_ receptors may vary according to their location in the central nervous system [21]. Upon activation by GABA, chloride ion influx results in hyperpolarization. This, in turn, leads to decreased postsynaptic neuronal excitability and suppresses the release of excitatory neurotransmitters. As such, inhibition of action potential generation can be attained [18,21]. Besides ethanol, the GABA_A_ receptor is also a target for several drugs, namely barbiturates, benzodiazepines, and general anaesthetic drugs [18].

Upon acute exposure, high blood concentrations of ethanol result in the potentiation of inhibitory GABA action at the GABA_A_ receptor, which confers central nervous system suppressive effects [20,23]. Studies have revealed that the increased frequency and prolongation of the opening duration of the chloride ion channel are among the factors contributing to the inhibitory action of ethanol. Effects of long-term exposure to ethanol were studied in animal models, and it was discovered that rats experiencing abstinence from ethanol (withdrawal) following chronic ethanol treatment showed greater susceptibility to seizure attacks in comparison to naive controls. It was also revealed that the degree of potentiation of GABAergic neurotransmission is greatly reduced in rats which underwent chronic ethanol treatment [20].

Being lipophilic [24], ethanol is able to pass through the phospholipid bilayer of cell membranes and affect several intracellular signalling proteins, particularly protein kinase C (PKC). Examination of brain tissues excised from mice, which lack PKCε and GABA receptors, has shown increased sensitivity to ethanol. For instance, 20 mmol/L of ethanol doubles GABAergic neurotransmission when compared to naïve controls [25]. On the other hand, the loss of ethanol-induced potentiation of GABAergic neurotransmission was observed in mutant mice where PKC-γ is absent [26]. Not only did these mice show increased ethanol consumption, but they also appeared to be more aggressive.

On top of that, studies discovered that certain brain regions of rats pre-sensitized by β-adrenoceptor agonists displayed ethanol-induced GABA_A_ receptor alteration. Therefore, it can be deduced that ethanol-induced potentiation of GABAergic neurotransmission requires high intracellular cyclic adenosine monophosphate levels, as well as high levels of PKA-mediated phosphorylation of intracellular protein sites following repeated ethanol administration [20].

### 2.2. Ethanol and NMDA Receptors

Glutamate, which is an excitatory neurotransmitter in the central nervous system, exerts its effects via three types of receptors, namely kainate, quisqualate, and NMDA receptors, which are responsible for postsynaptic signal transduction [27,28]. NMDA receptors, to which glutamate shows greater binding affinity, are heteromeric tetramers [29] formed from a combination of various subunits—NR1, NR2, or NR3. For instance, NMDA receptors could be made up of NR1 subunits that merge with one or more NR2 (A, B, C, D) subunits. NR3 subunits may be involved, but their significance is small when compared to NR2. NMDA receptors have been associated with various ethanol-related physiological and behavioural manifestations, such as tolerance, dependence, seizures, and so on. Upon chronic exposure to ethanol, the upregulation of NMDA receptors in certain brain regions is observed, to which seizure attacks during ethanol withdrawal can be attributed [28].

NMDA receptor-mediated calcium ion influx, which is responsible for receptor functionality, is implicated in synaptic plasticity, as well as action potential conductance. Examination of tissue samples excised from different brain regions, including the cerebral cortex, hippocampus [30], amygdala, nucleus accumbens [31], and dorsal striatum, are carried out to measure the magnitude of postsynaptic excitatory neurotransmission current to determine the inhibitory effects of ethanol. NR1-2B/NR2C combination is found to exhibit maximum inhibition by ethanol, whereas minimal inhibition is observed in NR1-3B/NR2C, NR-3B/NR2D, and NR1-4B/NR2C. Therefore, it can be proposed that the type of combination of NMDA receptor subunits is one of the factors affecting an individual’s sensitivity to ethanol [28].

### 2.3. Ethanol and Glycine Receptors

Glycine, being an inhibitory neurotransmitter, causes agonism of glycine receptors, primarily in the spinal cord and brainstem. This results in chloride ion influx, followed by hyperpolarization, which decreases postsynaptic neuronal excitability. At a concentration of 10mM, ethanol-induced potentiation of glycinergic currents is observed. It is proposed that the magnitude of postsynaptic current potentiation at glycine receptors is influenced by the positioning and organization of receptor subunits [32]. For instance, the α1 subunit plays an important role in the inhibitory phenomenon observed following the administration of ethanol [33,34]. On top of that, several studies have discovered that psychological effects following alcohol consumption, such as behavioural excitation as well as addiction [35], are regulated by glycine receptors [36,37].

Furthermore, animal models are used to determine the inhibitory action of glycinergic neurotransmission where mice with mutated α1 subunits, as well as β subunits, were studied. The studies have led to the discovery of hypertonia and other neurological disorders, such as hyperekplexia, which are similar to patients suffering from epilepsy (seizure attacks) [38,39]. Hence, it is postulated that genetic mutations are strongly linked to alterations of glycine receptor function.

Besides that, acute administration of ethanol can produce pleasurable experiences such as euphoria and behavioral excitation via activation of the mesolimbic dopaminergic system, which is also known as the reward reinforcement circuit of the brain. This results in increased levels of dopamine in the ventral tegmental area, nucleus accumbens, and amygdala in a dose-response fashion [24]. Due to the positioning at a lower level, dopamine release in the nucleus accumbens has been attributed to glycine receptors [35,40]. Moreover, increased dopamine levels in the nucleus accumbens following raised extracellular glycine levels can be seen after the glycine transporter type 1 (GlyT1) inhibitor is administered [41]. On the other hand, following chronic ethanol administration, microinjections of glycine into ventral tegmental area of rats are found to result in decreased ethanol consumption [42]. As such, glycine receptors can be made the targets for pharmacological treatment of alcohol intoxication, as well as addiction, where therapeutic agents that antagonize ethanol action on glycine receptors could be developed [32].

## 3. Structural Brain Alterations Associated with Ethanol Use

Higher brain functions including learning, memory, cognition, emotions, rational judgement, behaviour, as well as visuospatial impairment, have been observed in individuals with histories of chronic alcohol abuse [24,43]. Such higher brain function impairments have been attributed to alterations in brain structures. A myriad of findings obtained from non-invasive methods of magnetic resonance imaging and computed tomography, as well as physical and psychological studies on chronic alcohol abusers, show that the frontal lobe, the limbic system, and the cerebellum are especially susceptible to injury and destruction [44]. Although a significant correlation between cognitive impairment and structural loss of the brain is yet to be established, despite a wealth of evidence that has come to light, several impaired sensory and motor functions have been related to the loss of brain structures [43]. For instance, studies found that alcoholic individuals exhibit decreased olfactory function in relation to the change in volume of the thalamus, which is the odour-processing centre of the brain [45]. On the other hand, another study has led to the discovery of impaired ability to maintain postural balance and disorientation in alcoholics. This impaired coordination was found to be related to the volume of the anterior superior vermis [43]. Table 1 summarised the effects of acute/binge drinking and chronic alcohol consumption on different parts of the brain.

### 3.1. Effects on Frontal Lobe

The frontal lobe of the cerebral cortex has several important functions contributing to human qualities. It is involved in the planning of various goal-directed behaviours, the recognition of future consequences of current behaviour, the selection between good and bad actions, and the retention and modification of emotional memories derived from the limbic system to fit socially accepted norms. Besides that, the frontal lobe is deemed the moral centre of the brain, as it plays an important role in advanced decision-making processes [44].

Post-mortem studies of brain samples from alcoholics show an approximate 15 to 23% reduction in neuronal density in the frontal lobe of the cerebral cortex because of long-term ethanol exposure [44,46]. In addition, reduced volume, as well as dysfunction of the frontal lobe, are observed through functional magnetic resonance imaging studies. In cases of acute alcohol intoxication, a decreased rate of glucose metabolism in addition to reduced regional blood flow in the frontal lobe, are noted [47]. Physiological abnormalities were also noted prior to the discovery of any detectable brain atrophy and cognitive deficit [48].

Behavioural abnormalities related to frontal lobe structural and functional alterations are studied in chronic alcohol abusers with and without Korsakoff’s amnesic syndrome. Korsakoff’s amnesic syndrome is a late complication of persistent Wernicke encephalopathy, which is caused by thiamine (vitamin B1) deficiency [49]. It is characterised by memory deficits (retrograde and anterograde amnesia), mental confusion, and behavioural changes. It was later discovered that Korsakoff patients exhibit cognitive impairment, as well as amnesia and verbal fluency impairment, whereas non-Korsakoff patients display milder symptoms which are affected by several factors including age, duration of alcohol consumption, and withdrawal, as well as the amount consumed [44].

### 3.2. Effects on The Temporal Lobe

The temporal lobe of the brain, together with occipital lobe, the parietal lobe, and the frontal lobe, form the cerebral cortex. Located in the middle cranial fossa, the temporal lobe is involved in eight cognitive domains, namely speech perception and production, hearing, episodic memory, phonological processing, semantic processing, social processing, and visual processing [50].

Significant reductions in regional cerebral blood flow (rCBF) analysis on patients during alcohol withdrawal were observed compared to patients in a remitted state [51]. Significant temporal lobe volume deficits in cortical grey matter, white matter, and anterior hippocampus matter were observed in chronic alcoholic men by Sullivan et al. Following this observation, the same group further examined the relation between withdrawal seizure history and hippocampal or extrahippocampal volume deficits. It was found that the reduction of white matter volume deficits in the temporal lobe could lead to alcohol withdrawal seizures. Significant bilateral volume deficits of the temporal grey matter were observed in alcoholics who had one or more alcohol-related seizures and seizure-free alcoholics, with the seizure group having smaller temporal lobe white matter volumes comparatively [52]. Using deformation-based morphometry to examine brain structure differences in one-week-abstinent alcoholics and light drinkers, Cardenas et al. found that their cohort of predominantly male caucasians’ alcohol dependency was associated with a reduction in tissue in foci within the temporal lobes and frontal lobes [53].

Binge drinkers were reported to have higher electroencephalogram density of both beta and theta oscillations in the right temporal lobe and bilateral occipital cortex. This may result in difficulties in cognitive processing and decreased response to stimulation, often reflected by their inability to stop drinking. Moreover, deficits in episodic and verbal memory, impaired visual memory, vigilance, and sustained attention, which are associated with temporal lobe dysfunction, were noticeable in binge drinkers and women. These usually present worse cognitive plasticity compared to men due to their less-effective alcohol metabolism capabilities [54].

### 3.3. Effects on Limbic System

The limbic system, also known as the emotional nervous system, is comprised of numerous structures that encircle the brainstem. There is no defined list of structures making up the limbic system. In general, the limbic system consists of the cingulate gyrus, hippocampus, amygdala, anterior thalamus, and hypothalamus. The limbic system is involved in emotions, learning, memory, motivation, cognition, as well as feelings of pleasure and punishment [44,55].

The amygdala, with connections to both the hypothalamus and, indirectly, the prefrontal cortex, is involved in the processing, recognition, and interpretation of negative emotional behaviours and signals such as rage, sadness, and fear [56]. Dopamine, the main neurotransmitter that mediates the brain reward pathway, regulates feelings of pleasure and punishment, and it also partially exerts its effect on the amygdala [57]. A study was carried out on two groups of individuals—chronic alcohol abusers with alcohol abstinence and control participants. Both groups were presented with emotional words and facial expressions, followed by functional magnetic resonance imaging scan. A higher degree of amygdala activity was stimulated in the control group in comparison to chronic alcohol abusers, in which activation was less significant [44].

The hippocampus, which is also known as the memory centre of the brain, is in the temporal lobe. The hippocampal memory system of mammalian brain spans several brain regions in addition to the hippocampus, such as areas in the adjacent parahippocampal region and many distributed unimodal and polymodal neocortical areas. It is widely believed that the hippocampus plays a vital role in the formation of specific personal experience events (episodic memory), as well as their associated emotions [44,58].

Previous studies regarding the effect of ethanol on the formation, proliferation, and survival of neurons have been conducted using animal models. Ethanol is found to impair neurogenesis in the adult rat hippocampus via two modes: the proliferation and the survival of neurons. Ultimately, such alterations and disruptions result in cognitive impairments, similar to those observed in chronic alcohol abusers. In addition, decreased hippocampal volume is also observed in chronic alcohol abusers [59] following MRI scans, with the left side being more significant than the right one. This is mainly caused by white matter alterations [60]. Nevertheless, such observed volumetric reduction, as well as the associated cognitive impairments, were corrected following a short period of ethanol abstinence [61,62].

The hypothalamus, located in the most inferior portion of the diencephalon, houses several small nuclei and tracts. It is involved in a range of homeostatic regulatory functions, namely blood pressure regulation, pupillary dilation, neuroendocrine control, thirst and hunger, urinary bladder contraction, and so on. The negative impact of ethanol on hypothalamic function appears to be more significant in the mamillary body regulating feeding reflexes following chronic alcohol consumption and simultaneous dietary malnourishment. Subsequent manifestations of memory deficit will be observed, where the disorder is referred to as Korsakoff’s amnesic syndrome. Individuals with alcoholic Korsakoff’s amnesic syndrome are unable to make lasting memories of their daily experiences (anterograde amnesia). The preservation of memories is limited only to those which are formed before ethanol-induced brain damage. Besides that, Korsakoff’s amnesic patients also experience loss of muscle coordination (ataxia), violent behaviour, and confabulation [44,63].

Adolescents and young adults tend to binge drink compared to older adults, and they are more sensitive to the effects of alcohol. Their reward area was found to be matured earlier compared to the cognitive control cortex, which contributed to their decision making of consuming alcohol. Apart from that, higher dopamine release in the striatum, associated with a more rewarding system of binge drinking, was noticed in adolescents. Binge drinking in young adults also saw a reduction in their cortical thickness in pruning regions of the cortex, and they also have an increased risk of neurocognitive dysfunction coupled with hippocampus and entorhinal cortex degeneration [54].

### 3.4. Effects on Cerebellum

The cerebellum is almost exclusively involved in motor control. For instance, it regulates the coordination of skilled, voluntary movements such as eating a cake and playing a piano, adjusts posture and balance, and corrects errors of ongoing movements, all of which result in smooth and balanced muscular activity. In addition, the cerebellum is also involved in cognition, such as learning, memory, planning, and problem solving. The cerebellum is composed of four types of GABAergic neurons, which are Purkinje cells from which axons are projected out of the cerebellum, stellate cells, basket cells, and Golgi cells. Only one type of glutaminergic neuron is found in the cerebellum, which is the granule cell [44].

Shrinkage of the cerebellum is often linked to chronic alcohol abuse. A marked decrease in cerebellar vermis volume is noted in 25 to 40% of alcohol abusers. Besides that, a 42% volumetric reduction of vermal white matter [44,64] is observed in those experiencing disorientation of voluntary movements (ataxia). Furthermore, scientific evidence has led to the establishment of a meaningful relationship between cerebellar atrophy and executive functioning in relation to the frontal lobe, thereby reporting the significance of disarrayed frontocerebellar pathway in the cascade of ethanol-induced functional degradation of the brain [44,65]. Moreover, researchers suggested a compensatory mechanism maintaining proper functioning of the frontocerebellar pathway, where a rising urge may be provoked on the frontal lobe to vanquish ethanol-induced functional impairments, whereas the cerebellum plays a supplementary role in information retention to counteract brain system deterioration [65,66].

On top of that, chronic alcohol abusers suffering from Korsakoff’s amnesic syndrome have reported a marked slump in Purkinje cell density in the vermal region, as well as volumetric decrease in the molecular layer containing stellate and basket interneurons [64]. It is important to take note on the irreversibility of ethanol-induced cerebellar damage following ethanol withdrawal. The underlying mechanism stimulating a cascade of neurological and mitochondrial destructions after complete ethanol abstinence is reported to have involved the release of glutamate in the cerebellum where excitatory neurotransmission mediated by glutaminergic granule cells is projected to Purkinje cells. This results in a rise in cytosolic Ca^2+^ levels and a fall in Ca^2+^-binding proteins, followed by Ca^2+^ influx into the mitochondria. Subsequent delayed closure of mitochondrial permeability transition pore, and the excessive production of free radicals, ultimately impair the production of adenosine triphosphate (ATP) molecules. Thus, mitochondria can be a potential therapeutic target in the management of cerebellar dysfunction following ethanol withdrawal [67,68].

Dysfunction of the central nervous system was noticed in young binge drinkers, which was a negative impact of the direct action of ethanol and its metabolites (acetaldehyde, ROS, and methanol) on brain metabolism, electrical properties of the membranes, activation of microglia and immune response in the cerebellum, and immunogenicity of lipopolysaccharide. Noticeably, binge drinkers also have an increased volume in their cerebellum, which affects their risky decision-making abilities. A decreased fractional anisotropy ratio, the marker of white matter integrity, was also reported in the cerebellum of binge drinkers [54].

## 4. Structural Brain Alterations Associated with Ethanol Use in Animals

The use of structural magnetic resonance imaging to detect ventricular expansion in animal models of alcoholism has been consistent, with the timing and route of ethanol administration being the key two factors influencing the experimental outcome. In binge rat models fed nasogastric ethanol that induced an immediate surge in blood alcohol levels (BALs), ventricular size abnormalities were found to be more significant than in those who received long-term ethanol inhalation treatment [69]. For example, a 115% increase in ventricular volume was observed in heterogeneous stock Wistar rats placed under 3 g/kg intragastric binge ethanol treatment three times daily for four consecutive days, with BALs of 258 mg/dL as compared to basal levels [70]. Subsequently, they also reported a 122% increase in ventricular volume and average BALs of 292 mg/dL following binge ethanol treatment. This indicates a dose-response relationship between the amount of ethanol consumed and the degree of ventricular enlargement [71]. Besides that, N-acetylaspartate (NAA) is lower and choline-containing compound (Cho) is higher in these groups of rats [70,71]. In contrast to what has been observed in humans, similar changes have been shown to be self-limiting, as ventricular volume and brain neurochemicals reverted to normal size and levels following ethanol cessation for less than a week. Even if the binge ethanol treatment was repeated for another five cycles, despite gradual ventricular expansion during each cycle, ventriculomegaly still appeared to be transient because the ventricles reverted to normal volume for each period of cessation [72]. On the other hand, inhalation ethanol treatment for a period of six months yielded only a 30% increase in ventricular volume, with BALs of 444 mg/dL being attained [73]. In chronic rodent models, lower levels of NAA and total creatinine (tCr) were negligible, whereas Cho levels increased in a dose-dependent manner [74]. This evidence suggests that acute and chronic ethanol treatment via distinct routes of administration triggers different adaptation mechanisms.

While there remain questions about the underlying mechanism of ventriculomegaly, Zahr and colleagues conducted research to see how strain differences among rats affect morphological changes in the brain related with binge ethanol therapy [75]. Three groups of rats consisting of 11 Wistar rats (W), 12 alcohol-preferring (P) rats, and 12 nonalcohol-preferring (NP) rats were given an initial intragastric loading dose of 5 g/kg 25% *w/v* ethanol, followed by 3 g/kg, three times daily, for four consecutive days. In comparison to both W and NP rats, ethanol-naive P rats have larger ventricles. To put it another way, P rats’ brains may be smaller in specific regions. Zhou and colleagues supported this hypothesis, reporting lower serotonergic innervations in the hippocampus, nucleus accumbens, and cortex [76], as well as lower dopamine levels in the selective cingulum cortex and nucleus accumbens shell of the medial mesolimbic system [77]. In addition, Miguel-Hidalgo’s group also reported lower astrocyte packing density in the prelimbic cortex [78]. Similarly, analysis between Marchigian-Sardinian P rats and outbred Wistar rats revealed congenital smaller grey matter volumes in the dorsal lateral thalamus, lateral ventral tegmental area, substantia nigra, anterior and posterior cingulate (retrosplenial), and insular cortices in the former groups of rats, which might constitute their vulnerability to ethanol abuse and dependence identical to those observed in alcoholics [79]. Similar to prior research, the current study found that binge ethanol treatment resulted in a significant increase in ventricular capacity in Wistar rats than basal and recovery levels, as compared to a 5% dextrose pair-fed control group. In P rats, no such alterations were observed. During binge treatment, P rats in both the ethanol and control groups developed ventriculomegaly. Binge ethanol-treated NP rats, on the other hand, showed reversible ventriculomegaly with 70% volumetric expansion when compared to basal and recovery levels. However, there were no treatment differences during the three phases within the time frame of baseline, binge, and recovery, where NP rats of the control group recorded identical ventricular volume as those binge-treated with ethanol. Considering the degree of ventriculomegaly in binge ethanol-treated NP rats was smaller than Wistar and P rats receiving equivalent dosing, NP rats may be more resistant towards structural brain alterations due to high BALs. As a result, marked ventriculomegaly after binge ethanol administration appears to be a Wistar rat-specific phenotype. As opposed to the unique effects of binge ethanol therapy on ventricles, all three groups of rats had lower binge NAA and tCr levels than basal and recovery levels, but Glu levels remained unchanged, reflecting a consistent overall response. Only Wistar rats showed substantial ventriculomegaly and Cho elevations among the three groups of binge ethanol-treated rats, indicating a relationship between these two variables at both the basal and binge levels. Cho increases were minor in P rats, but the increase in NP rats was considered significant because the control group showed a dip between basal and binge levels [75].

Surprisingly, structural brain alterations associated with ethanol use were discovered to be age-related. Cognitive dysfunction was observed in adult rats subjected to both an acute paradigm of 5 g/kg ethanol and a chronic paradigm of 9.3 g/kg/day divided into three doses over a four-day period because of ethanol’s potent inhibitory action on progenitor cell proliferation and neurogenesis in the hippocampus [80]. Similar findings were made in adolescent rats as well [81]. A 14-day diet of 4% *w/v* liquid ethanol in adult rats resulted in a 40% decrease in hippocampal neurogenesis regardless of gender [82]. Nevertheless, the adolescent rat brain is more prone to the toxic effects of binge ethanol treatment. Adolescent rats exposed to inhalation ethanol had ventriculomegaly, as well as hippocampal atrophy, because of decreased progenitor cell proliferation and neurogenesis, which persisted as they matured into adults [83,84], as opposed to reversible inhibition seen in adult rats [85]. Another study, conducted by Coleman et al., where C57BL/6J mice aged 28 to 37 days post-natal days were gavage fed 5 g/kg/day of 25% *w/v* ethanol once daily for 10 days as a binge rodent model, was considered. On day 79 post-natal, post-mortem MRI automatic segmentation analysis revealed a volumetric loss of 6.5% and 4.4% in the olfactory bulb and basal forebrain, respectively [86].

## 5. Oxidative Stress and Anxiety

While a large body of data have shown that GABAergic and serotonergic neurotransmission abnormalities are involved in the pathophysiology of anxiety disorders, several studies have suggested that oxidative stress may play a role. Kuloglu and his colleagues (2002) identified a connection between oxidative stress and anxiety disorders, including panic disorder and obsessive-compulsive disorder (OCD). In comparison to healthy controls, individuals with anxiety disorders were reported to show increased levels of oxidative stress indicators such as glutathione peroxidase, superoxide dismutase (SOD), catalase (CAT), and malondialdehyde (MDA) in their blood samples [87]. Since then, the idea that oxidative stress plays a role in anxiety has prompted researchers to dig more into the veracity of this theory. Later, in 2005, Hovatta and associates studied the relation between antioxidant system activity and anxiety symptoms using antioxidative metabolic enzymes such glyoxalase 1 and glutathione reductase 1 as criteria in six inbred mouse strains. Via genetic manipulation using lentivirus-mediated gene transfer, they discovered that levels of enzymatic activity in the brains of these transgenic mice are directly proportional to anxiety-related phenotypes, with the most anxious mice having the highest level of enzymatic activity and the least anxious mice having the lowest level of enzymatic activity. Furthermore, more anxious mice had local overexpression of both genes in the cingulate cortex of their brains, whereas less anxious mice had RNA interference that resulted in local suppression of glyoxalase 1 expression. As a result, the regulatory function of both enzymes in the anxiety phenotype remains conceivable [88]. Brain proteomic analysis in several brain areas was conducted on two Swiss CD1 mice strains bred for high-anxiety-related behaviour (HAB-M) or low-anxiety-related behaviour (LAB-M) that exhibit different anxiety-like behavioural characteristics. According to these findings, lower anxiety phenotypes had higher levels of glyoxalase 1 expression than higher anxiety phenotypes [89,90]. These findings contradicted those of Hovatta and colleagues [88]. Differences in the genotypes of the mouse strains may be partly to blame for the variance in their findings. Nevertheless, findings of these researchers suggested that glyoxalase 1 might be a promising biological marker for anxiety phenotype.

In support of Hovatta et al.’s preliminary findings, Bouayed and partners used 2′,7′-dichlorofluorescein diacetate to evaluate peripheral oxidative status in leukocytes of Swiss albino male mice with or without anxiety and found more reactive oxygen species (ROS) being generated in highly anxious mice compared to those without anxiety [91]. The presence of ROS in the brains of these mice were studied further using a behavioural light/dark choice test, which revealed that highly anxious mice had considerably greater levels of ROS in the cerebellar and hippocampal neurons and neuroglia, as well as cerebral cortical neurons. In peripheral blood, phenotype-dependent variations were also detected [92].

Phosphodiesterase-2 (PDE2) is a ubiquitous enzyme that is involved in the regulation of intracellular anti-inflammatory cAMP and/or cGMP signalling by hydrolytic reaction. Being highly abundant in several brain regions, its unique characteristic leads its blockage to result in enhanced cGMP or cAMP signalling, which may aid in inhibiting ROS generation, restoring intracellular redox status, and alleviating neuropsychiatric diseases such as depression and anxiety [93,94]. Masood and collaborators evaluated the effect of inhibition of PDE2 in reversing oxidative stress-induced anxiety in mouse models. In their study, oxidative stress was induced in male ICR mice through daily intraperitoneal administration of 300 mg/kg L-buthionine-(S,R)-sulfoximine (BSO) for two consecutive days prior to behavioural testing. PDE2 inhibitor (3 mg/kg Bay 60-7550), NADPH oxidase inhibitor (3 mg/kg apocynin), and 1 mg/kg diazepam were administered via similar route 30 min prior to BSO administration. BSO-treated mice exhibited anxiogenic behaviour in elevated plus maze, hole-board, and open field tests. Interestingly, this was successfully reversed through inhibition of PDE2 and NADPH oxidase by Bay 60-7550 and apocynin respectively, whereas diazepam had no effect. Higher levels of lipid peroxides reduced total antioxidant capacity as well as increased expression of NADPH oxidase subunits and generation of superoxide anions/ROS were observed in the amygdala and hypothalamus, brain regions involved in anxiety following BSO treatment. However, these conditions were reversed by PDE2 inhibitor Bay 60-7550, which boosts cGMP-protein kinase G (PKG) signalling by increasing phosphorylation of VASP at Ser239 in the amygdala and hypothalamus, as confirmed by Western blotting. In addition, Bay 60-7550 also suppresses NADPH oxidase pathway that produces oxidative stress. Similar improvement was observed for apocynin too. However, it is important to understand that apocynin does not affect cGMP-PKG signalling, as it does not affect p-VASP^Ser239^ expression. Instead, it works by directly inhibiting the NADPH oxidase enzyme, which is responsible for superoxide anion and ROS production. Considering hypothalamic-pituitary-adrenal (HPA) axis disruptions and subsequent corticosterone level alterations from oxidative stress that are involved in various neuropsychiatric disorders, inhibition of PDE2 or NADPH oxidase may mitigate oxidative stress-induced oxidative stress and anxiogenesis by acting either directly or indirectly on the axis. Being an anxiolytic drug class of benzodiazepine, diazepam, which works by potentiating GABAergic neuronal signalling, did not appear to be fully effective in the management of oxidative stress-induced anxiety [95].

## 6. Oxidative Stress and Depression

Following a 40-day chronic variable stress (CVS) treatment, Tagliari and colleagues observed increased lipid peroxidation, as assessed by an increase in thiobarbituric acid reactive species levels, as well as decreased SOD levels in male Wistar rat models, both of which implied oxidative stress [96]. Subsequently, Morgana and collaborators reported antioxidant and neuroprotective effects of ascorbic acid on chronic unpredictable stress (CUS)-induced depression in female Swiss mice. Their findings added to the growing body of evidence supporting the role of oxidative stress in the pathophysiology of depression. In their study, mice were subjected to daily CUS for two weeks. Starting from day eight of CUS treatment, daily 10 mg/kg ascorbic acid or fluoxetine were given orally to the mice. At the end of the study, depression was observed in vehicle-fed stressed mice where longer immobility time was recorded in a tail suspension test and less time spent on grooming was seen in a splash test. On the contrary, stressed mice given ascorbic acid or fluoxetine showed a substantial decrease in depressive-like behaviour. CUS-induced depressive-like behaviour was associated with increased levels of TBARS, which implied increased lipid peroxidation in the cerebral cortex and hippocampus. Furthermore, decreased glutathione levels in the cerebral cortex, as well as decreased cerebral cortical and hippocampal CAT and hippocampal glutathione reductase (GR) activities, were reported. Stressed mice receiving daily ascorbic acid and fluoxetine showed favourable improvement in depression and intracellular redox status. No alterations in locomotion were observed in an open field test. Corticosterone levels and cerebral cortical and hippocampal glutathione peroxidase (GPx) activities were all unaffected. These experimental findings show that ascorbic acid could correct neuropsychological and biochemical alterations caused by stress-induced oxidative damage in mice, indicating that this vitamin might be a viable option for treating depressed symptoms [97]. The beneficial effects of ascorbic acid were consolidated by Mostafa and associates. Paediatric patients who received 1000 mg daily ascorbic acid supplement and 10–20 mg daily fluoxetine treatment for six months, concurrently, demonstrated a substantial reduction in depressive symptoms as evidenced by improvement in the Children’s Depression Rating Scale (t = 11.36, *p* < 0.0001) and Children’s Depression Inventory (t = 12.27, *p* < 0.0001) scores compared to those who were treated with fluoxetine only. Clinical Global Impression, on the other hand, revealed no significant alterations (t = 0.13, *p* = 0.90) [98].

## 7. Alcohol Use Disorder, Oxidative Stress and Psychological Disorders

Psychiatric disorder usually occurs simultaneously with alcohol use disorder (AUD), with depression being the most prevalent [99,100]. AUD is characterized by persistent binge alcohol intake while aware of its negative impacts. Individuals suffering from alcohol-induced depression would experience emotional distress and depressive symptoms during the course of alcohol intoxication or withdrawal, where their condition usually alleviates following about one month of complete abstinence [99].

Despite scientific evidence indicating the correlation between simultaneous occurrence of AUD and depression, the precise aetiology of ethanol-induced depression is still yet to be determined [99]. Genetic predisposition, as well as oxidative stress, have been identified as risk factors [99,101]. Recent studies conducted by researchers have conceptualized a marked relationship between inflammation and oxidative stress, in addition to neurodegeneration [101]. The mechanism of alcohol-related oxidative stress involves microsomal and mitochondrial metabolism of ethanol, which exposes the brain to oxidative stress through the generation of harmful reactive oxygen species (ROS), in addition to reactive nitrogen species (RNS) [102,103]. Susceptibility of the brain to damage by oxidative stress can be attributed to its higher oxygen consumption, higher lipid content, and weaker antioxidative defence [104]. Although low to moderate amounts of ROS is vital for neurogenesis, when produced in amounts beyond detoxification capacity of the antioxidant system, cellular apoptosis activation occurs, causing irreversible cellular and tissue damage. Such loss of dynamic equilibrium between ROS production and depletion of antioxidative defences, such as glutathione, can result in neuropsychiatric disorder due to extensive protein oxidation and lipid peroxidation, as well as impaired glutamate-mediated long-term potentiation of memory formation [102]. Levels of MDA generated from lipid peroxidation of polyunsaturated fatty acids [105], as well as ROS-generated nitric oxide [102], are found to be elevated in alcoholics that also experience depression [106].

## 8. Ethanol Withdrawal

Alcohol withdrawal syndrome (AWS) comprises a broad spectrum of clinical presentations that manifests following reduced intake, or even complete abstinence, from alcohol. During alcohol withdrawal, removal of neurotransmission inhibition mediated by GABA [18,21], as well as upregulation and activation of NMDA receptors, result in brain hyperactivity such as seizure attacks, autonomic activation manifested as a sudden surge in blood pressure and body temperature, hyperventilation, diaphoresis (heavy perspiration), and tachycardia [28,107].

The intensity of AWS is influenced by the degree of alcohol dependence as well as duration of alcohol abuse. For instance, the longer the duration of alcohol abuse, the more intense the AWS symptoms experienced by the alcohol abuser. In some cases, a severe form of AWS known as delirium tremens (DT) may set in [108,109]. According to New England Journal of Medicine (NEJM), approximately 3 to 5 percent of chronic alcohol abusers will experience DT [110], characterized by generalized tonic-clonic seizure, which is caused by abnormal and synchronized neuronal firing in the brain [111], in addition to delirium (mental confusion with lowered awareness to one’s surroundings) and circulatory failure [110]. The onset of DT will be triggered approximately two days after abrupt, complete alcohol withdrawal and can persist for up to five days. DT is a medical emergency, which requires immediate medical attention for prompt treatment. Should there be any delay in delivering treatment, a death rate of up to 37% can be expected [110].

Interestingly, there are no proven medications to treat AWS. Treatment of AWS often involves symptomatic management. For example, benzodiazepines, such as lorazepam and diazepam, will be used for their anxiolytic and hypnotic effects [109]. Besides that, nutrition replenishment can be attained by taking vitamin supplements such as thiamine and folic acid [112]. In addition, eating a well-balanced diet rich in fruits and vegetables and maintenance of adequate hydration are important, as well, to rejuvenate the body [109].

### 8.1. Neurobiology of Ethanol Withdrawal

Being multicellular organisms, humans have an internal regulatory mechanism known as homeostasis, which seeks to maintain a relatively constant internal environment for the optimum functions of cells. For instance, to maintain a stable body temperature of 37 °C, the integumentary system, nervous system, circulatory system, muscular system, and endocrine system work in a manner such that a rise or a drop in temperature above or below 37 °C would result in a cascade of pathways to adjust the physical and chemical factors to their optimum range via vasodilation and vasoconstriction, in addition to other mechanisms [113]. Tolerance is a phenomenon that occurs when chronic exposure to a certain substance, which in this context is ethanol, results in physiological neuro-adaptation process which is pharmacologically known as dependence, where up-regulation of CNS receptors can be observed. As such, dose escalation is required to achieve the same pharmacological effect as seen in those elicited in naïve users. Neuroadaptation causes compulsive ethanol seeking behaviour to readjust the brain to a state of excitation and this constitutes a vicious cycle of ethanol addiction, as a person would find him/herself experiencing both physical and psychological disorders such as pain, discomfort, and anxiety when going through a period of abstinence (negative reinforcement) following chronic ethanol abuse. Ethanol addiction generally involves two modes, namely, positive and negative reinforcements. For instance, when a person consumes ethanol to achieve a feeling of “high” and euphoria for a pleasurable experience, this phenomenon is known as positive reinforcement. However, when the person consumes ethanol to avoid experiencing unbearable physical and psychological discomfort, the phenomenon would be otherwise known as negative reinforcement, and this is how a person seeking for casual pleasure ultimately becomes an ethanol addict. These neuroadaptive changes precipitate alcohol withdrawal symptoms following abrupt alcohol cessation, and it is widely accepted that anxiety experienced during ethanol withdrawal is the culprit causing relapse to ethanol abuse [114,115,116].

Interestingly, several human and animal models have successfully yielded scientific evidence, which came to light to demonstrate a correlation between the endogenous opioid system, which is involved in regulation of ethanol consumption, and manifestation of ethanol addiction. For instance, acute ethanol consumption may stimulate brain reward circuitry which gives rise to euphoria via release of opioid peptides. Conversely, chronic ethanol consumption may cause central opioid deficiency, placing the individual in a state of withdrawal which, in turn, leads to ethanol craving. Three distinct families of classical opioid peptides have been identified, namely, enkephalins, endorphins, and dynorphins. Each family is derived from a distinct precursor protein and has a characteristic anatomical distribution. These precursors, proenkephalin, pro-opiomelanocortin (POMC), and prodynorphin, respectively, are encoded by three corresponding genes. Proenkephalin produces methionine enkephalin (met-ENK) and leucine-enkephalin (leu-ENK), and both demonstrate a 10-to-25-fold increase in binding affinity towards δ opioid receptors. β-endorphin, which is derived from POMC [117], is involved in pain management which, when being injected directly into the brain, becomes a more potent analgesic than morphine [118]. It functions primarily as a μ-opioid receptor agonist which contributes to its analgesic property, in addition to a similar degree of δ action [117]. Interestingly, its localisation in the hypothalamus renders it a neurohormone, which can modulate the release of other hormones involved in the regulation of sexual functioning and maternal breast feeding [118]. For example, it decreases the release of luteinizing hormone (LH) and follicle stimulating hormone (FSH) and increases the release of growth hormone [119] and prolactin. Dynorphin, which is responsible for regulation of pain at the spinal and supraspinal levels, appears to be more potent on ҡ-opioid receptors [117]. μ-opioid receptors are thought to be responsible for most of the analgesic effects of opioids and accountable for unwanted adverse effects of opioids analgesics, such as respiratory depression and the euphoria associated with morphine use. On the other hand, δ-opioid receptors are probably more important in the periphery but are believed to contribute to analgesia as well. Moreover, ҡ-opioid receptors are responsible for analgesia at the spinal level and may elicit sedation and dysphoria but produce relatively less adverse effects and do not contribute to dependence. All opioid receptors are G-protein coupled receptors, which upon binding of an opioid agonist, such as morphine, would result in inhibition of adenylyl cyclase, thereby reducing intracellular cAMP levels. In addition, they stimulate the opening of K^+^ channels and inhibit opening of voltage-gated Ca^2+^ channels, resulting in hyperpolarisation, reduced neurotransmitter release, reduced neuronal excitability, and, ultimately, reduced pain conductance [120].

The brain reward circuitry, which produces pleasurable feelings when stimulated, includes the ventral tegmental area (VTA), from which dopaminergic neurons are mainly projected to NAc, in addition to olfactory tubercle innervating the septal area, amygdala, and hippocampus. Opioid receptors residing in the VTA and NAc regulate dopamine release following both acute and chronic ethanol consumption, where compulsive ethanol seeking behaviour can be attributed. Researchers have suggested that ethanol-induced behavioural and endocrinological disorders, as well as reinforcement, are due to changes in the activity of endogenous opioid peptides [117]. Even though all three classical opioid peptides are involved in the process, effects of ethanol on hypothalamic and pituitary secretion of β-endorphin have received the most attention. In an in vivo study conducted by Thiagarajan et al., acute administration of a single dose of 12% ethanol (3.2 g/kg) into adult male rats has led to an increased release of hypothalamic corticotropin releasing hormone (CRH) and activation of hypothalamic-pituitary-adrenal axis, which is indicated by an increase in adrenocorticotropic hormone (ACTH) and corticosterone (CS), as well as a concomitant surge in plasma levels of adrenaline and β-endorphin [121]. Moreover, alcohol-preferring C57BL/6 and alcohol-avoiding DBA/2 mice have been utilized to determine the effects of various concentrations of ethanol on their hypothalamic β-endorphin release. Findings suggested that the alcohol preferring C57BL/6 mice exhibits greater hypothalamic β-endorphin release. Upon treatment with 10, 20, and 25 mM of ethanol, a marked rise in hypothalamic β-endorphin release was observed in both strains of mice. However, when treated with 30 and 60 mM of ethanol, the increase was not as significant, all of which produces an inverse U-shaped dose-response curve. Genetic predisposition plays a role in affecting the experimental outcome as well, wherein mRNA for POMC is found to be higher in the hypothalamus of alcohol-preferring C57BL/6 mice and is able to affect their voluntary alcohol consuming behaviour [122]. This study was extrapolated to determine the effect of single and repeated ethanol exposure on hypothalamic β-endorphin release. It was later deduced that ethanol-induced hypothalamic β-endorphin release is not sustainable. It lasts around 15 to 20 min, followed by a gradual fall back to baseline. Further ethanol exposure following 30 min of recovery period showed no increased release of β-endorphin. A prolongation of the recovery period to 60 min would, again, result in increased hypothalamic β-endorphin release in alcohol preferring C57BL/6 mice [123].

Nevertheless, the effects of chronic ethanol exposure on β-endorphin release by the pituitary gland and the hypothalamus remain inconclusive. While some studies suggested an increased β-endorphin biosynthesis, others suggested otherwise. Gianoulakis et al. reported an increased biosynthesis of β-endorphins in neurointermediate pituitary lobes of male Sprague Dawley rats, which was indicated by increased in vitro incorporation of [3H] phenylalanine into POMC, β-lipotropin, and β-endorphin on day zero of ethanol withdrawal following a 21 day 6.5% *v/v* ethanol liquid diet. This was also recorded on days one and three, but no profound difference was observed on days 8 and 15. On the other hand, both ethanol and sucrose (control) fed rats displayed a higher rate of in vitro incorporation of [3H] phenylalanine into POMC, β-lipotropin, and β-endorphin on days 8 and 15, following ethanol withdrawal, in comparison to day zero, thereby proposing the significance of nutritional factors. Additionally, the HPLC of β-endorphins revealed a greater amount of acetylated β-endorphins in the neurointermediate lobes of ethanol fed rats, with the increase being most significant on days 8 and 15 following ethanol cessation. As such, the assumption that, although biosynthesis and secretion of β-endorphins return to their normal rate of functioning on day 15 following ethanol treatment, high activity of β-endorphin acetylating enzyme remains plausible [124]. In another study, Seizinger et al. placed rats under chronic treatment with a 15% *v/v* ethanol liquid diet for a period of 21 days. The outcomes discovered a significant 50% slump in neurointermediate pituitary secretion of β-endorphin both in vivo and in vitro. Analysis using SDS-PAGE suggested a decrease in in vitro biosynthesis of POMC and subsequent production of β-endorphin due to a delay in post-translational modification process. As such, development of ethanol tolerance is thought to be the underlying reason for inconsistencies in these findings [125].

On top of that, in another study conducted by Scanlon, two groups of rats were placed under ethanol and water vapour (control) inhalation treatment for a period of 10 days. Upon examination, while pituitary POMC mRNA level in ethanol treated rats remained unaffected, decreased hypothalamic POMC mRNA level was reported, which in turn led to a corresponding decrease in POMC expression [126]. However, one year later, this finding was contradicted by a study conducted by Gianoulakis et al., where immunoprecipitation and SDS PAGE analysis revealed higher levels of POMC mRNA in the hypothalamus of rats chronically treated with ethanol for 15 days, resulting in a corresponding increase in POMC expression [127]. Variations in study findings are likely caused by duration and dose of ethanol treatment, as well as the route of administration. Besides that, the contradiction that arose may also be due to different species and/or strains of mice being employed in the study, as well as development of ethanol tolerance [117].

In a nutshell, an increased β-endorphinergic activity does not necessarily mediate voluntary ethanol consumption. However, a decreased β-endorphinergic activity following chronic ethanol exposure may exert negative reinforcement effects. After consuming ethanol for an extended period, maintenance of a relatively normal and stable CNS functionality is attained via neuroadaptation. As such, upon ethanol cessation, the CNS finds itself in a deficiency state, which produces an urge for ethanol supply to compensate the neurotransmitter (dopamine) shortage. This, in turn, results in craving behaviour as the person consumes ethanol continuously to relieve discomfort or pain during ethanol withdrawal.

In comparison to β-endorphins, only a few studies were carried out on enkephalins and dynorphins. However, the effects of ethanol on these two opioid peptides were found to be affected by the species, strain, and line of animal models [117]. For instance, Nylander et al. carried out a study on the mesolimbic reward brain circuitry of two rat lines, namely alcohol-preferring (AA) rats and alcohol-avoiding (ANA) rats. Comparative study reported a lower basal level of (Met)enkephalinArg6Phe7 (MEAP) and dynorphin peptides in the nucleus accumbens of AA rats, as well as lower (Leu)enkephalinArg6 levels in their ventral tegmental area. However, ethanol treatment had resulted in increased MEAP levels in the nucleus accumbens without having profound effect on prodynorphin peptides [128]. Later, in the year of 2000, with the utilization of in situ hybridization, Marinelli et al. reported a greater abundance of proenkephalin mRNA in the prefrontal cortex of AA rats than in ANA rats, and the same was observed in the mediodorsal nucleus of the thalamus, where prodynorphin mRNA is harboured [129]. In another animal study using C57BL/6J and DBA/2J mouse models, similar levels of enkephalin gene expression can be seen in the hypothalamus, striatum, hippocampus, and medulla pons of both strains, whereas lower content of proenkephalin mRNA and the corresponding reduced gene expression was discovered in the midbrain of the C57BL/6J mice compared to that in DBA/2J mice. Following 17 g/kg/day ethanol treatment, C57BL/6J mice demonstrated a significant increase in enkephalin peptide levels in the striatum and mid brain. Hence, it can be proposed that organisms with inherent low basal levels of enkephalin are more prone to ethanol abuse, wherein the latter would result in amplified mesolimbic enkephalin production [130]. Interestingly, the greater abundance of pro-dynorphin mRNA and dynorphin peptides in DBA/2J mice may have contributed to their nature of ethanol avoidance [131]. The inconclusiveness of previous research findings suggests that future research should be directed towards resolving confusion regarding complex interaction between alterations of the endogenous opioid system and ethanol consumption.

While it is suggested that ethanol-induced behavioural changes, such as ethanol reinforcement, are also due to alterations of activity, as well as affinity of opioid receptors for their ligands, the results are diverse considering that the type of opioid receptors, in addition to other physiological variations including blood alcohol concentration and route of administration, as well as the species of animal models, differ among researchers. For example, previous studies carried out on brain membrane specimens with acute ethanol treatment recorded a reduced binding affinity of receptor ligands to δ, whereas such observation was not seen in μ or κ opioid receptors. Meanwhile, other researchers had recorded an increased binding affinity to μ opioid receptors. On top of that, following chronic ethanol treatment, a reduced binding affinity was observed for δ opioid receptors, but binding affinity on μ opioid receptors remains an area of interest among researchers to reach a universal agreement [117]. Thanks to the rapid innovation of scientific technologies, studies of ethanol on CNS opioid receptor pathways using more specific ligands were made possible especially with autoradiographic techniques. In the year 1999, Fadda et al. carried out an experiment to study various components of the opioid system scattered in specific brain regions of two types of rats, namely Sardinian alcohol-preferring rats (sP) and Sardinian alcohol non-preferring rats (sNP) for a period of 30 days. The experiment recorded an increased in ligand binding to both μ and δ opioid receptors in the caudate putamen, whereas such improvement was not seen in other brain regions [132]. In the same year, another pre-clinical study was carried out by Turchan et al., where Wistar rats were fed with ethanol-infused drinking water at concentrations rising from 1 to 6% gradually for a period of one month. The study resulted in decreased density of μ opioid receptors in the nucleus accumbens and striatum, whereas such downregulation was not observed for δ_1_ and δ_2_ opioid receptors [133]. Hence, it can be deduced that species of animal models are one of the factors that would affect study outcomes regarding ethanol-induced opioid receptor system alterations.

Current pharmacological treatment of ethanol dependence uses naloxone, as well as naltrexone, which are nonspecific opioid receptor antagonists, capable of displacing opioid receptor agonists from the receptor sites. Binding and the subsequent blocking effects on opioid receptors can reduce pleasurable rewarding sensation following ethanol consumption and compulsive ethanol seeking behaviour stimulated by environmental factors in a dose-response fashion. As such, it greatly reduces relapse to ethanol abuse [117]. Validation using animal models has shown a drop in dopamine levels in the nucleus accumbens, which subsequently results in reduced ethanol consumption. For instance, Heyser et al. studied the effect of methylnaloxonium administration on ethanol self-administration in rats via oral route. In this study, male Wistar rats underwent a 30 min training in a limited access paradigm daily to observe how they respond to 10% *w/v* ethanol or water. Methylnaloxonium was then injected into the amygdala and nucleus accumbens of the rats, where their voluntary self-administration behaviour was studied 15 min later. They discovered that 250–500 ng of methylnaloxonium, when injected into the amygdala, resulted in a decrease response to ethanol, whereas 500–1000 ng injection into the nucleus accumbens was required for the same effect. These findings suggested that opioid receptors scattered in those two brain regions might play a role in controlling ethanol self-administration [134].

Naltrexone shows a good and favourable safety profile and is generally well-tolerated. As such, naltrexone is approved for use in treating ethanol dependence and addiction in conjunction with other non-pharmacological measures such as psychosocial counselling and cognitive behavioural therapy [135,136]. In a nutshell, a thorough understanding of interactions among constituents of the opioid receptor system, as well as with others which are involved in alcoholism, as well as ethanol-induced behavioural alterations, may yield a satisfactory finding in the development of therapeutic agent for alcoholism treatment.

### 8.2. Kindling Hypothesis: Role of Anticonvulsants

The kindling hypothesis suggests that a person deprived of a substance of abuse, most commonly ethanol and benzodiazepines, would experience withdrawal symptoms, with each subsequent episode being worse than the previous one. The construction of the kindling concept is based on epilepsy animal models, which correspond to the link between the frequency of ethanol withdrawal episodes and the intensity of withdrawal symptoms. In other words, one of the most prominent characteristics of ethanol withdrawal, which is seizure, is manifested because of CNS hyperexcitability due to neurotransmitter sensitization, particularly glutamate-induced excitotoxicity [137]. Clinical studies using mouse models revealed that mice which underwent repeated ethanol withdrawal episodes are more prone to seizures in comparison to those which underwent single ethanol withdrawal episode, whereby an exaggerated epileptogenic phenomenon has been observed following consecutive withdrawal events. This was supported by clinical findings from a study conducted by Becker et al. In her study, three groups of adult C3H mice of multiple withdrawal (MW), single withdrawal (SW), and control underwent chronic treatment with ethanol vapor. During the first phase, both MW and SW mice were given multiple and single session of ethanol treatment prior to testing, with the first MW group of mice being placed under nine, six, or three cycles of 16 h continuous ethanol treatment with an intermediate of 8 h abstinence, whereas the SW group of mice were placed under a single cycle of 16 h of continuous ethanol treatment without interruption. The control group of mice remained naïve and was not exposed to ethanol during experiment. The experiment proceeded by testing MW1-9 mice which had undergone nine cycles of ethanol withdrawal. Pyrazole was also given repeatedly to determine whether it can affect withdrawal-induced kindling. The study outcomes revealed a proportional correlation between the number of ethanol withdrawal cycles and the intensity of withdrawal-induced seizure. Similar blood ethanol levels were recorded in mice which underwent ethanol treatment before withdrawal examination. Also, pyrazole was found to have no effect on withdrawal-induced seizures [138]. The same situation was observed in human models, as well, thus leading to the solidification of the kindling hypothesis.

Although the characterization of the exact mechanisms underlying kindling remains a subject of interest, induction of NMDA and GABA receptor alterations because of recurrent ethanol withdrawal episodes has been proposed. Upon activation, NMDA receptor-mediated calcium ion influx results in excitatory glutaminergic neurotransmission. It has been proven, in animal studies, that acute ethanol consumption would exert inhibitory effects on NMDA receptors. Meanwhile, prolonged excitatory neurotransmission inhibition, due to chronic ethanol consumption, would result in an increased density of NMDA receptors in several brain regions, where this occurrence is known as upregulation. As such, ethanol withdrawal would result in increased glutamate release, as well as increased excitatory neurotransmission, due to excessive Ca^2+^ influx, all of which ultimately result in excitotoxicity and withdrawal seizure. On the other hand, the potentiation of GABAergic neurotransmission upon acute exposure to ethanol would result in Cl^-^ influx and subsequent hyperpolarization. As such, inhibition of action potential generation is achieved. However, upon chronic ethanol exposure, downregulation of GABA_A_ receptors takes place, resulting in decreased potentiation of GABAergic neurotransmission [137].

Etifoxine has been studied to exploit its applications in the management of ethanol withdrawal, owing to its anxiolytic and anticonvulsant characteristics, while being superior to benzodiazepine as it does not cause sedation and ataxia. By potentiating GABA inhibitory neurotransmission, its benefits were proven in 2009 by Marc Verleye. In his experiment, NMRI mice were fed with 3% ethanol for a period of 8 days, then subsequently raised the concentration to 4% for another week, which gave a dose ranging from 24 to 30 g/kg to induce withdrawal symptoms following abrupt cessation of ethanol treatment. Three and a half hours after ethanol cessation, intraperitoneal injection of etifoxine (12.5–25 mg/kg) and diazepam (1–4 mg/kg) were given successfully, and they decreased handling-induced tremors and seizures, as seen 4 to 6 h post-withdrawal. On top of that, administration of both etifoxine (50 mg/kg) and diazepam (1 mg/kg) 30 and 15 min prior to light and dark box tests was shown to prevent events of unpleasant withdrawal symptoms 8 h post-withdrawal. At doses of 25 and 50 mg/kg, etifoxine had no effect on locomotor activity as well as ataxia [139].

The development of pharmacological management of alcohol withdrawal has led to exploitation of benzodiazepines and other antiepileptic drugs for their potential neuroprotective effects. Nevertheless, antiepileptics have been proven useful for their ability to shield against withdrawal seizure, thus improving the ethanol withdrawal experience. For instance, Na^+^ channel blockers, such as oxcarbazepine and lamotrigine, inhibit voltage-gated Na^+^ channels in glutaminergic neurons, thereby inhibiting depolarization and subsequent Ca^2+^ influx via voltage-gated Ca^2+^ channels. On the other hand, gabapentin decreases Ca^2+^ influx by binding to α2δ-1 subunit of Ca^2+^ channels, which is involved in the regulation of Ca^2+^ influx. It is important to take note of the significance of gabapentin for both its anticonvulsive as well as its anxiogenic properties, as its validation has been carried out using rodent models [137]. In a double-blinded randomized clinical trial conducted by Myrick and his colleagues, gabapentin and lorazepam treatment were given to alcoholics experiencing withdrawal symptoms for a period of four days with gradual dose tapering. The study proved the safety and superiority in efficacy of gabapentin in these patients compared to those receiving lorazepam [140]. Since antiepileptics work by decreasing excitatory glutamate neurotransmission, they are deemed promising therapeutic agents in the management of withdrawal seizure. However, further clinical studies on the validation of correlation between ethanol, antiepileptics, and neurotransmission cascade using animal and human models are yet to be carried out for a more thorough understanding of ethanol withdrawal kindling.

On top of this, antiepileptics, which work by increasing inhibitory GABAergic neurotransmission, are also employed in the effort to supress seizures seen during ethanol withdrawal. Valproate, having a dual mode of action, not only acts as a Na^+^ channel blocker, but it also inhibits GABA transaminase and prevents GABA degradation in presynaptic neurons [141]. This results in a favourable increase in GABA storage for the future release and subsequent decrease in postsynaptic neuronal firing due to increased GABA binding. Besides that, topiramate acts as a GABA_A_ receptor agonist, where it binds and activates GABA_A_ receptors in a manner similar to endogenous ligands (GABA), thus triggering Cl^−^ influx and depolarization [137].

Several mouse models were used for kindling studies using benzodiazepines as therapeutic agents. For example, mice vulnerable to intensified withdrawal symptoms were exposed to continuous or chronic intermittent ethanol treatment (CIE). One group was placed under four cycles of 16 h continuous ethanol treatment with an intermediate of 8 h abstinence, whereas the other two groups were placed under four cycles of 16 h continuous ethanol treatment without period of abstinence and act as naïve controls, respectively. An electroencephalogram (EEG) for these three groups of mice was then evaluated for the elucidation of brain electrical activity, where comparison can then be made with respect to the use of different types of therapeutic agents for their effects on both single and repeated ethanol withdrawal episodes. In one study which employed this mouse model, mice were administered with lorazepam with an increasing dose of 0.25–1.0 mg/kg. It was revealed that the alleviation of sensitized handling-induced convulsions in the acute phase of unmedicated withdrawal events occurred in a dose-dependent manner. However, as the experiment proceeded, aggravation of withdrawal-induced seizure was observed, which can be attributed to lorazepam withdrawal [142]. The fact that neuronal hyperexcitability is specific to particular brain regions has captured the interest of researchers. Dating back to year 1996, Veatch and Gonzalez examined the brain electrical activity from different brain regions of male Sprague-Dawley rats using EEG following 10 to 20 days of chronic ethanol treatment with a period of four days of abstinence in between. Following cessation of ethanol treatment, random measurement of EEG was conducted for three days. The hippocampal regions recorded the first change in spike and sharp wave activity, with area CA1 being most significantly affected by alterations in the amount of ethanol exposure and area CA3 being most markedly influenced by the number of withdrawal cycles. Following further ethanol treatment, a higher level of activity was seen in the cortical and subcortical areas [143].

Diazepam demonstrated a suppressive effect on acute withdrawal symptoms. However, when administered during intermittent withdrawal, it showed no raised seizure threshold during later unmedicated withdrawal [144]. This experimental outcome proposed that benzodiazepines, when used in the management of acute withdrawal symptoms, neither confer neuroprotection nor alleviate withdrawal seizure following repeated withdrawal episodes. Last, but not least, carbamazepine has emerged as a promising therapeutic agent for improvement of withdrawal seizure in patients experiencing mild to moderate withdrawal symptoms [136]. In 2010, Barrons and his colleagues evaluated carbamazepine, as well as oxcarbazepine, for their safety and efficacy in the management of withdrawal seizures. Their study concluded that carbamazepine has a desirable safety profile, and it is effective in treating moderate to severe withdrawal symptoms. However, there was a lack of evidence supporting the use of carbamazepine for its prophylactic effect on withdrawal seizure and delirium tremens. Benzodiazepines remain the drug of choice for the management of moderate to severe withdrawal symptoms [145]. With enormous efforts being placed in the development of pharmacological management of alcohol withdrawal syndrome, it is expected that the general well-being of alcohol abusers undergoing detoxification can be assured with their unpleasant withdrawal symptoms under good control by various safe and effective medications, in addition to the risk of relapse being minimized.

## 9. Ethanol Withdrawal, Corticotropin-Releasing Factor, and Neurological Disorders

Corticotropin-releasing factor (CRF), also known as corticotropin-releasing hormone (CRH), is a hypothalamic neuropeptide hormone consisting of 41 amino acid residues that is involved in stress regulation. Activation of the hypothalamic-pituitary-adrenal axis occurs when CRF is secreted by CRF-positive neurons in the paraventricular nucleus of the hypothalamus in response to stress (such as during ethanol withdrawal) or low blood glucose levels. CRF passes through the hypothalamohypophyseal portal system to the anterior pituitary gland, where it activates CRF receptor type 1 (CRF_1_) in the anterior pituitary, which results in secretion of adrenocorticotropic hormone (ACTH). ACTH, in turn, stimulates secretion of glucocorticoids (cortisol in humans and corticosterone by rodents) in the zona fasciculata of adrenal glands by binding to their receptors on the adrenal cortex [146]. Findings from recent years discovered that CRF is not only confined to the hypothalamus, but it also exists in extrahypothalamic structures such as the central nucleus of the amygdala (CeA) as well as in the bed nucleus of stria terminalis (BNST), in addition to the brainstem. Besides that, extrahypothalamic CRF is suggested to be involved in the regulation of behavioural stress and anxiety disorders. Interestingly, while endocrine stress triggers CRF binding to pituitary CRF_1_ receptors, amygdala and BNST CRF_1_ receptors are those that mediate behavioural stress [147].

Ethanol withdrawal produces an urge for ethanol craving, a condition known as negative reinforcement in ethanol-dependent subjects to avoid experiencing aversive stimuli arising from painful withdrawal symptoms, with elevated anxiety being one of the hallmark features. Increased CRF release in the CeA and heightened CRF signalling within the amygdala are reported to underly the mechanism for this phenomenon. In humans, during ethanol withdrawal, elevated anxiety state is experienced during the early phase, and there is a high chance this anxiety would persist for a long time [148]. CRF_1_ receptor upregulation in the amygdala has been reported following ethanol dependence, and CRF receptor antagonists is able to prevent withdrawal anxiety and associated increased voluntary ethanol consumption. Interestingly, the majority of CeA neurons in rodents are GABAergic, which project inhibitory neurotransmission to brainstem nuclei. Previous studies reported increased GABAergic neurotransmission by CRF and ethanol which is mediated by CRF_1_ receptors. However, it remains a question of how exactly GABA neurotransmission in the CeA affects CRF and ethanol-associated behavioural and motivational alterations [149]. It is also important to take note that raised plasma levels of adrenaline and noradrenaline due to autonomic sympathetic activation are responsible for tachycardia, hypertension, tremor, restlessness, and other symptoms of withdrawal. During the early stage of ethanol withdrawal, increased CSF levels of noradrenaline were observed, thereby indicating noradrenergic activation, which is associated with increased diastolic blood pressure [150,151].

Baldwin et al. conducted an experiment to study the relation between CRF, anxiety, and ethanol withdrawal in male Wistar rats. In the first group of drug naïve control, rats received intracerebroventricular CRF administration of 0, 0.1, and 0.5 μcg CRF. On the other hand, the CRF antagonist, alpha-helical CRF of doses of 0, 5, 25 and 50 μcg, was administered to the other group of mice. An elevated plus maze test revealed that 0.5 μcg CRF produces anxiety-like behaviour, as well as reduced locomotor activity, seen as decreased time spent, as well as the number of entries onto the open arms, while 5 and 25 μcg of alpha-helical CRF showed no attenuation of the rats’ behaviour. At the maximum dose of 50 μcg, CRF-like activity exhibited is not statistically significant. In the second group of studies, 12 to 15 rats were assigned to each group of different alpha-helical CRF doses—0, 5 and 25 μcg, respectively. They reported an anxiety-like behaviour and reduced activity following 8 h of ethanol withdrawal in the behavioural paradigm for rats, which received 14 to 21 days of 8.5–11.5% *v/v* ethanol liquid diet in comparison to sucrose pair-fed rats. The anxiogenic behaviour is characterized by decreased time spent, as well as the number of entries onto the open arms, where intracerebroventricular administration of the CRF antagonist, alpha-helical CRF, was shown to reverse the behaviour aforementioned. However, the occurrence of tail stiffness, body tremor, and ventromedial distal flexion remained unaffected. Hence, it can be deduced that the antagonism effect of alpha-helical CRF on clinical manifestations of ethanol withdrawal does not occur by a simple opposite mechanism. Instead, it only antagonizes anxiety behaviour following ethanol cessation. Also, the attenuation of anxiogenic responses by intracerebroventricular administration of alpha-helical CRF suggested that CeA is the centre of CRF-mediated withdrawal anxiety [152]. Similar findings were reported by Funk et al., as well. In their experiment, the non-selective CRF antagonist D-Phe-CRF_12–41_ was administered into the CeA of both ethanol-dependent and non-dependent rats, to which ethanol-dependent rats showed reduced post-dependent voluntary ethanol consumption, whereas non-dependent rats showed no observable response. On the other hand, administration into the lateral BNST and the nucleus accumbens shell was ineffective in both groups of rats. These data demonstrate that CRF, particularly in the CeA, is the site of regulation of uncontrollable ethanol consumption following withdrawal in alcoholics [153]. They also reported excessive voluntary consumption in ethanol-dependent rats following 2 h of ethanol cessation, to which systemic administration of non-peptide CRF_1_ antagonists, namely antalarmin, MJL-1–109–2, and R121919, successfully brought down CRF to basal levels. However, the antagonists were ineffective in non-dependent rats [153].

The role of CRF in ethanol withdrawal-induced anxiety was further validated by Overstreet et al. [154]. In this study, male Sprague Dawley rats were employed as the animal models. Rats that received intracerebroventricular administration of 1 μg CRF displayed lower level of social interaction compared to those that received artificial cerebrospinal fluid (CSF) as a vehicle, thereby implying the anxiogenic effect of CRF. Furthermore, pre-treatment with CRF one day before 7% *v/v* ethanol liquid diet for a period of five days displayed a marked reduction in social interaction compared to those pre-treated with artificial CSF on ethanol liquid diet, as well as in those that are ethanol naïve. This suggested that CRF sensitized ethanol withdrawal-induced anxiety. Also, the effects of CRF_1_ receptor antagonist CRA 1000 were found to be able to reverse ethanol withdrawal-induced anxiety. In this investigation, one group of rats fed with a control diet for 15 days served as control, and another three groups of rats which underwent three cycles of 5 days 7% *w/v* ethanol liquid diet with two days of ethanol abstinence between the three cycles were treated with CRA1000 (CRF_1_ receptor antagonist, 4 h after ethanol withdrawal during first and second cycles) and another group with carboxymethylcellulose (CMC). The fourth group of rats received 1 mg/kg of CRA 1000 30 min prior to social interaction test or 4.5 h following cessation of the third cycle of ethanol treatment. While control group rats displayed more active social interaction than CMC-treated ethanol exposed rats, rats receiving CRA1000 at the same time as CMC behaved similarly to control rats. CRA1000 given after final withdrawal did not appear to attenuate social behaviour of the rats. The same improvement was observed, as well, in rats treated with 10 mg/kg of nonpeptide CRF_1_ antagonist CP-154,526 during first and second withdrawal. These findings suggested that the CRF_1_ receptor antagonist does not act as an anxiolytic itself. Rather, it antagonizes the anxiogenic effect of CRF. However, 20 μg of CRF_2_ antagonist antisauvagine-30 did not appear to protect against withdrawal-induced anxiety. As such, it can be assumed that CRF_2_ receptors do not contribute to the sensitization of ethanol withdrawal-induced anxiety. Nevertheless, this remains inconclusive since only a single dose was administered [155]. Also, it is also possible that CRF_2_ may produce anxiety since Spina et al. had reported anxiogenic effects of urocortin, which shows great binding affinity towards CRF_2_ receptors [155].

Several rodent studies have demonstrated the regulatory role of CRF in stress-induced reinstatement of post-dependent ethanol consumption behaviour. By utilizing the footshock stressor behavioural paradigm, the D-Phe CRF_12–41_ (0.3 or 1 μg) and CRF_1_ selective antagonist CP-154 526 (15, 30 or 45 mg/kg) dose-dependently prevented stress-induced relapse to ethanol drinking. In addition, the regulatory role of extrahypothalamic CRF system in behavioural stress was validated via adrenalectomy where adrenal glands were removed to eliminate endogenous corticosterone. Neither adrenalectomy, nor 50 mg/kg per day corticosterone supplementation, caused stress-induced relapse to ethanol drinking [156]. D-Phe CRF_12–41_ can only prevent stress-induced relapsing behaviour, whereas cue-induced relapsing behaviour is prevented by the opioid receptor antagonist naltrexone [157]. Footshock stress-induced relapsing behaviour can also be attenuated by direct injection of D-Phe CRF_12–41_ into the median raphe nucleus [158].

While these research findings point to a correlation between oxidative stress and anxiety disorders, a causal relationship remains unaddressed. Only an association could be made, while the underlying mechanism remains a mystery. While the technique of establishing the anxiogenic impact of oxidative stress has significant limitations, the current evidence supports this causal connection. Antioxidants may be of relevance because of the putative causative involvement of oxidative stress in anxiety. Last, but not least, the ongoing development of promising novel therapeutic agents should be motivated to bring a glimpse of hope to society for the management of negative emotional states experienced by alcoholics during withdrawal so that their general well-being can be assured.

## 10. Brain-Derived Neurotropic Factor, Alcohol Disorders and Depression

An association between depression and suicide with structural and synaptic plasticity has been established. Brain-derived neurotrophic factor (BDNF) is a homodimeric protein, and its expression in the adult mammalian brain has proven its importance in neurogenesis, neurotransmission, and synaptic plasticity, particularly in brain regions regulating emotions and cognition such as the hippocampus, the prefrontal cortex, the nucleus accumbens, and the amygdala via activation of its tyrosine kinase receptor B (TrkB) [159,160]. As such, pathological disruptions of BDNF may constitute the pathophysiology of neuropsychiatric disorders, such as depression, and result in behavioural abnormalities. Much scientific evidence has revealed lower levels of BDNF expression in depressed patients, as well as those who exhibit suicidal tendencies, though both events may not be directly linked to each other [161]. Roni and Rahman have reported disruption of BDNF expression in the dentate gyrus and CA3 region of the hippocampus in animals which abstained from chronic ethanol exposure [162]. BDNF, also implicated in alcohol addiction, was reported to be low in rats under ethanol withdrawal by Ghitza et al. [163]. This was also seen in the study by Hou et al., where significantly lower levels of protein BDNF were seen in the hippocampus and nucleus accumbens of the ethanol group after withdrawal compared to the control after withdrawal [164]. A recent study found that Z-guggulsterone, derived from the gum resin of *Commiphora mukul*, could activate the BDNF signalling cascade, as well as increase hippocampal neurogenesis, thus producing antidepressant-like effect [165]. Genetics may play a role in the development of pathological alterations of BDNF expression. For example, single nucleotide polymorphism (SNP) in the BDNF gene, whereby valine residue is replaced with methionine residue in codon 66 (Val66Met), would ultimately impair neuronal activity-mediated BDNF secretion [166] and cause the person to be more vulnerable to depression [167]. Hippocampal atrophy has been reported in individuals with Val66Met SNP, which causes them to have impaired hippocampal function [168].

Being part of the limbic system, the hippocampus plays a vital role in the formation of specific personal experience events (episodic memory), as well as associated emotions [44,58]. Besides that, it can regulate the inhibition of hypothalamic–pituitary–adrenal (HPA) axis activity that mediates systemic stress responses. Volumetric studies on hippocampal atrophy have been conducted, and they revealed a 4 to 5% reduction in hippocampal volume in depressed patients in comparison to healthy controls [169]. An investigation was carried out to study the association of depression with hippocampal atrophy by Bauer et al., which showed significant signs of cellular suicide (apoptosis) in in situ DNA end-labelling of hippocampal tissues obtained from depressed patients, particularly in the dentate gyrus, CA1, and CA4 areas [170]. On top of that, 18% shrinkage in hippocampal tissues of depressed patients have been noted with the aid of neuroimaging [171]. Interestingly, greater pre-treatment hippocampal volumes were seen in depressive patients in remission with treatment than those who were not [172]. In addition, compared to healthy controls, depressed patients with smaller left and right hippocampal volumes had a higher chance of recurrence [173]. With the support of a plethora of evidence, researchers are confident that hippocampal apoptosis and atrophy are among the signs of depression. Since BDNF is important for neurogenesis, hippocampal atrophy may be attributed to lower BDNF levels, as seen in depressed patients. As mentioned earlier, suicidal patients have low levels of BDNF as well. A post-mortem study on the Brodmann area (BA) 9 and the hippocampus, obtained from individuals who committed suicide, was conducted in 2003 by Dwivedi et al., and they reported low BDNF levels, as well as TrkB and cyclic adenosine monophosphate (cAMP) response element binding protein (CREB) [174]. Interestingly, immunohistochemistry shows that individuals under antidepressant treatment who committed suicide have higher BDNF levels in their dentate gyrus, hilus, and supragranular regions in comparison to antidepressant naïve individuals [175].

The effects of various classes of antidepressants on BDNF production have sparked the interest of researchers. For instance, antidepressants of selective serotonin reuptake inhibitor (SSRI) and noradrenaline reuptake inhibitor (NaRI) classes were used in a study conducted by Alejandre and colleagues to determine the effects of noradrenergic and serotonergic activation paired with physical exercise on BDNF expression in the hippocampus of depressed rat subjects. Rats under 40 mg/kg/day reboxetine treatment displayed a rapid rise in BDNF levels in several regions of their hippocampus, with the changes being most significant on day two of treatment. The same improvement was observed, as well, in rats receiving reboxetine treatment with a concomitant wheel running exercise for a period of 14 days. However, for those who received 10 mg/kg/day citalopram, only CA2 region displayed increased BDNF levels following two days of treatment. Its positive effect on BDNF expression appeared to require longer duration of treatment than reboxetine, where 14 days treatment paired with wheel running result in increased BDNF expression in the CA4 region as well as the dentate gyrus [176]. On the other hand, Nibuya and colleague utilized in situ hybridization and the Northern blot technique to determine the effects of electroconvulsive seizure (ECS) and antidepressants on BDNF and TrkB expression in rats’ brain. The study reported that rats which underwent 10 days ECS pre-treatment displayed a two-fold increase in BDNF expression in the frontal cortex upon acute ECS treatment. Besides that, chronic ECS treatment resulted in a marked induction of BDNF gene, as well as extension of BDNF and TrkB mRNA expression in CA3 and CA1 pyramidal cell layers. Furthermore, chronic antidepressant treatment for a period of 21 days with tranylcypromine, sertraline, desipramine, and mianserin had reported higher abundance of BDNF, whereas mianserin resulted in increased TrkB expression in the rats’ hippocampus. Therefore, it is said that ECS, as well as antidepressants, can block stress-induced BDNF expression dysfunction in the hippocampus, which may confer neuroprotection [177].

On top of that, the role of BDNF in contributing to the therapeutic efficacy of synthetic antidepressants is further supported by evidence provided by Shirayama et al., where direct intrahippocampal BDNF administration into dentate gyrus of rats resulted in antidepressant effect on day three of treatment, and the effect lasted for at least 10 days [178]. Heightened therapeutic response to paroxetine in a rat behavioural despair model was also established by Deltheil et al. in 2008. However, when the TrkB antagonist K252a is administered, the antidepressant effect of BDNF is diminished, thereby suggesting BDNF effect is TrkB-dependant, and that serotonergic activation is required for BDNF to exert its antidepressant effect [179]. Previous studies have demonstrated the link between increased BDNF expression and positive therapeutic outcomes of antidepressants. In the year 2010, Schmidt and colleagues investigated the potential functional significance of serum BDNF in various behaviour despair models of male C57Bl/6 mice to assess their depression- and anxiety-like behaviour. Their research findings reported that subcutaneous administration of BDNF in the periphery can produce antidepressant effect where mice under BDNF treatment displayed increased mobility in a forced swim test in a dose dependent manner after receiving 4, 8, and 12 μg per day of BDNF at a rate of 25 μL/h. In addition, post-mortem studies using enzyme-linked immunosorbent assay and in situ hybridization revealed a significantly heightened BDNF expression in mice hippocampus following 8 μg per day of peripheral BDNF administration for 14 days when compared to 0.9% *w/v* saline-treated controls, as well as improved survival of immature neurons in the dentate gyrus and hippocampus of mice. Similar outcomes were noted in mice receiving intraperitoneal injection of 100 mg/kg 5-bromo-2-deoxyuridine twice daily for two days prior to peripheral subcutaneous administration of BDNF. Meanwhile, the BDNF protein levels are found to be of not much difference in the ventral striatum between BDNF-treated mice and saline-treated controls. Surprisingly, the increases in BDNF mRNA levels are region specific. For instance, BDNF-treated mice showed a marked increase in BDNF mRNA levels in the CA3 region, but the difference is much less significant in the CA1 region or the dentate gyrus. However, BDNF did not affect the general locomotor activity of mice. In the open field test evaluation, no obvious change in the locomotor activity of mice was observed following peripheral 8 μg per day peripheral BDNF administration. This could be caused by different degrees of stress compared to that experienced by those placed in an elevated plus maze test, where BDNF displayed its anxiolytic role [180]. Although these data suggest that peripheral BDNF has antidepressant-like central activity, which confers therapeutic potential in the treatment of depression, the question of its clinical efficacy remains questionable considering the mechanism of its transport across the blood brain barrier is inconclusive [181,182,183]. The inadequacy of pharmacokinetic profiles, such as poor parenteral bioavailability and possible promotion of multiple myeloma progression [184], also limits the use of peripheral BDNF in treating depression.

The effects of BDNF on depression is specific to its target site. While it possesses antidepressant activity in the hippocampus, induced depression has been observed in VTA. For example, observation of rats placed under the forced swim test revealed that intra-VTA administration of BDNF led to a 57% increase in swimming, indicating the manifestation of depression, whereas disruption of BDNF neurotransmission due to truncated TrkB receptor in NAc results in a five-fold increase in time to reach immobility, indicating antidepressant effect [185]. Infusion of BDNF into the midbrain would result in monoaminergic activation as well, thereby conferring antidepressant-like behaviour. Learned helplessness test reported treatment groups of midbrain BDNF-infused rats with a daily dose of 12 to 24 μg, both with and without pre-treatment with inescapable shock can reverse escape impairment, as seen in vehicle-infused rats pre-treated with inescapable shock. Later, in the forced swim test, BDNF-infused rats displayed increased swimming time, therefore indicating the role of BDNF-induced monoaminergic activation in exhibiting antidepressant characteristic [186]. Although required in higher doses in order to achieved desirable clinical response [159], antidepressant-like behaviour induced by BDNF showed faster onset [178] and is longer lasting than synthetic antidepressants [187].

Since oxidative stress was proposed as one of the causes of depression, and because lower levels of BDNF are linked to emotional despair, Hacioglu and collaborators studied the neuroprotective effect of BDNF on stress-induced oxidative damage in transgenic male mouse models. BDNF heterozygous mice subjected to 2 h of the acute restraint stress procedure displayed greater vulnerability to stress and brain lipid peroxidation, as evidenced by higher levels of corticosterone and malondialdehyde (MDA), which are biomarkers of stress and lipid peroxidation, respectively, in comparison to stressed wild type mice. Besides that, the increment in activity levels of both antioxidative enzymes SOD and CAT was more significant in stressed wild type mice, indicating that BDNF confers tolerance to stress-induced oxidative damage. However, the enzymatic activity of SOD was comparable across study groups, which could be largely attributed to the detrimental effects of corticosterone on the ROS-scavenging capacity of the brain [188].

## 11. Ethanol Withdrawal, Oxidative Stress, and Psychological Disorders

The rationale behind the development of ethanol withdrawal symptoms has been accepted to be due to increase in excitatory glutamatergic and reduced inhibitory GABAergic neurotransmission in the central nervous system. While oxidative stress has been hypothesised, its role was not definitive, as conflicting studies have been reported. Nevertheless, a study by Parthasarathy et al. in 2015 showed that there were changes in the oxidative stress parameters during the withdrawal and remission of withdrawal phases. During the withdrawal phase, higher malondialdehyde and SOD levels were reported in the group of participants with alcohol withdrawal symptoms, while their CAT levels were lower than the control group [189]. Elevation of plasma thiobarbituric acid-reacting substances (TBARS) levels and vascular generation of superoxide anion were noted in male Wistar rats treated with 3–9% *v/v* ethanol for 21 days and abruptly discontinued to induce withdrawal [190]. However, Assis and colleagues did not find any differences in the TBARS levels in their experimental animals using the same method as Gonzaga above, but changes were noted in concentration of hydrogen peroxide (reduced) and CAT activity (increased) in the left ventricles of the animals [191]. Despite contradicting observations, it could not be denied that, during the phase of ethanol withdrawal, there are changes in the oxidative stress status, but this requires more in-depth studies to fully consolidate this hypothesis.

## 12. Pharmacotherapy for Ethanol Use Disorders-Associated Psychological Disorders

As ethanol induces brain neurotransmitter changes that might exacerbate depression, antidepressant medicines have been the mainstay of pharmacotherapy for alcoholics with comorbid depression. A slew of meta-analyses had shown that antidepressants are more efficacious than placebo in reducing depressive symptoms in patients with comorbid ethanol use disorder. The efficacy of antidepressants is comparable in depressive patients both with and without ethanol use disorder [192,193]. Considering most researchers placed more focus on placebo-controlled randomized trials, the relative effectiveness of different antidepressants has yet to be determined [194]. Despite this, it has been claimed that tricyclic antidepressants (TCA) are preferable to selective serotonin reuptake inhibitors (SSRIs) in the treatment of depression [192,195]. The lower efficacy of SSRIs may be due to the higher placebo response found in SSRI studies [193].

Antidepressants, interestingly, have just a slight influence on ethanol use [192,195]. Their impacts on depression may substantially influence how they regulate ethanol use. According to data reported by Quitkin and colleagues, depression appears to modify the influence of antidepressants on drinking outcomes [196]. Furthermore, Torrens and colleagues did a meta-analysis of antidepressant intervention in alcoholics without concomitant depression and found no significant differences in ethanol drinking behaviour between those who received antidepressants and those who were given placebo [195].

Petrakis and collaborators reported that naltrexone and disulfiram were effective in reducing ethanol craving and improving mood among patients with comorbid ethanol use disorder and major depression. Reduced ethanol consumption may have contributed to the remission of depressive symptoms [197]. On top of that, the efficacy of acamprosate treatment in reducing ethanol craving appeared to be comparable among alcoholics both with and without concomitant depression, and abstinence from alcohol was found to have a positive impact on the mood outcomes of depressive alcoholics [198]. Last, but not least, combination therapy, such as sertraline with naltrexone and acamprosate with escitalopram, has been shown to improve both drinking and mood outcomes in depressed alcoholics [199,200].

## 13. Conclusions

Alcohol abuse and alcohol withdrawal both could lead to neuropsychological problems such as depression and anxiety. It is widely accepted that this is due to the changes observed in the central nervous system. Although there are differences in the observed individual oxidative stress parameters studied, it is generally agreed on that alcohol abuse and alcohol withdrawal could exacerbate oxidative stress, and this has been linked to depression and anxiety. Even though these neuropsychological disorders could be controlled by antidepressants, their effects may not be sustainable, and their effectiveness may vary between individuals. With the numerous studies conducted on the neuroprotective effect of vitamin E, it is believed that vitamin E could be a potential treatment option in alleviating depression and anxiety disorders. Nevertheless, further studies are still needed, as the underlying mechanism of neuropsychological disorders induced by alcohol abuse and alcohol withdrawal is multifaceted.

## Figures and Tables

**Table 1:** The effects of acute/binge drinking and chronic alcohol consumptions on the brain.

Structure	Alcohol Consumption	
	Acute/Binge Drinking	Chronic
Frontal lobe	Decreased rate of glucose metabolism Decreased regional blood flow plus physiological abnormalities	Reduced neuronal density in post-mortem samples Reduced volume of frontal lobe Dysfunction
Temporal lobe	Increased electroencephalogram density of both beta and theta oscillations in right temporal lobe and bilateral occipital cortex → difficulty in cognitive processing and reduced response to stimulation Reduced episodic and verbal memory, impaired vision, vigilance, and sustained attention	Reduced volume in cortical grey and white matter and the anterior hippocampus Reduced tissue in foci in continuous light drinkers
Limbic systems	Increased dopamine release in striatum Reduced cortical thickness in pruning regions of the cortex Increased risk of neurocognitive dysfunction coupled with hippocampus and entorhinal cortex dysfunction	Altered and disrupted proliferation and survival of neurons → cognitive impairments Decreased hippocampal volume that is more prominent in the left side than the right (correctable after a period of abstinence) Negatively impacted mamillary body that regulates feeding reflexes of the hypothalamus → memory deficit Reduced amygdala activity stimulation → reduced ability to recognise and interpret negative emotions
Cerebellum	Central nervous dysfunction Increased volume Reduced fractional anisotropy ratio, marker of white matter integrity	Decreased cerebellar vermis volume Reduced vermal white matter in those experiencing ataxia Reduced Purkinje cell density in the vermal region and volumetric decrease in molecular layer containing stellate and basket interneurons in chronic abusers with Korsakoff’s amnesic syndrome

## Data Availability

Not applicable.

## References

[1] Alcohol. Definition, Formula, & Facts. Britannica. https://www.britannica.com/science/alcohol.

[2] Harmful Use of Alcohol. https://www.who.int/health-topics/alcohol#tab=tab_1.

[3] Sudan Scraps Apostasy Law and Alcohol Ban for Non-Muslims. BBC News. https://www.bbc.com/news/world-africa-53379733.

[4] Witkiewitz K., Litten R.Z., Leggio L. (2019). Advances in the science and treatment of alcohol use disorder. Sci. Adv..

[5] Drinking Too Much Alcohol Can Harm Your Health. Learn the Facts. CDC. https://www.cdc.gov/alcohol/fact-sheets/alcohol-use.htm.

[6] Zipursky J.S., Stall N.M., Silverstein W.K., Huang Q., Chau J., Hillmer M.P., Redelmeier D.A. (2021). Alcohol Sales and Alcohol-Related Emergencies during the COVID-19 Pandemic. Ann. Intern. Med..

[7] ICD-11 for Mortality and Morbidity Statistics. WHO. https://icd.who.int/browse11/l-m/en.

[8] Murthy P., Narasimha V.L. (2021). Effects of the COVID-19 pandemic and lockdown on alcohol use disorders and complications. Curr. Opin. Psychiatry.

[9] Mukamal K.J., Robbins J.A., Cauley J.A., Kern L.M., Siscovick D.S. (2007). Alcohol consumption, bone density, and hip fracture among older adults: The cardiovascular health study. Osteoporos. Int..

[10] Brust J.C. (2010). Ethanol and cognition: Indirect effects, neurotoxicity and neuroprotection: A review. Int. J. Environ. Res. Public Health.

[11] Osna N.A., Donohue T.M., Kharbanda K.K. (2017). Alcoholic Liver Disease: Pathogenesis and Current Management. Alcohol Res..

[12] Pöschl G., Seitz H.K. (2004). Alcohol and cancer. Alcohol Alcohol..

[13] Allen N.E., Beral V., Casabonne D., Kan S.W., Reeves G.K., Brown A., Green J. (2009). Moderate Alcohol Intake and Cancer Incidence in Women. J. Natl. Cancer Inst..

[14] Cao Y., Willett W., Rimm E.B., Stampfer M.J., Giovannucci E.L. (2015). Light to moderate intake of alcohol, drinking patterns, and risk of cancer: Results from two prospective US cohort studies. BMJ.

[15] Lundsberg L.S., Pensak M.J., Gariepy A.M. (2020). Is Periconceptional Substance Use Associated with Unintended Pregnancy?. Womens Health Rep..

[16] Mostofsky E., Mukamal K.J., Giovannucci E.L., Stampfer M.J., Rimm E.B. (2016). Key Findings on Alcohol Consumption and a Variety of Health Outcomes from the Nurses’ Health Study. Am. J. Public Health.

[17] Mukherjee R.A., Hollins S., Turk J. (2006). Fetal alcohol spectrum disorder: An overview. J. R. Soc. Med..

[18] Lobo I.A., Harris R.A. (2008). GABA(A) receptors and alcohol. Pharmacol. Biochem. Behav..

[19] Harris R.A., Trudell J.R., Mihic S.J. (2008). Ethanol’s molecular targets. Sci. Signal..

[20] Davies M. (2003). The role of GABAA receptors in mediating the effects of alcohol in the central nervous system. J. Psychiatry Neurosci..

[21] Edwards Z., Preuss C.V. (2022). GABA Receptor Positive Allosteric Modulators.

[22] Goetz T., Arslan A., Wisden W., Wulff P., Tepper J.M., Abercrombie E.D., Bolam J.P. (2007). GABAA receptors: Structure and function in the basal ganglia. Gaba and the Basal Ganglia.

[23] Mukherjee S. (2013). Alcoholism and its effects on the central nervous system. Curr. Neurovasc. Res..

[24] Ma H., Zhu G. (2014). The dopamine system and alcohol dependence. Shanghai Arch. Psychiatry.

[25] Hodge C.W., Mehmert K.K., Kelley S.P., McMahon T., Haywood A., Olive M.F., Wang D., Sanchez-Perez A.M., Messing R.O. (1999). Supersensitivity to allosteric GABA(A) receptor modulators and alcohol in mice lacking PKCε. Nat. Neurosci..

[26] Harris R.A., McQuilkin S.J., Paylor R., Abeliovich A., Tonegawa S., Wehner J.M. (1995). Mutant mice lacking the γ isoform of protein kinase C show decreased behavioral actions of ethanol and altered function of γ-aminobutyrate type A receptors. Proc. Natl. Acad. Sci. USA.

[27] Hoffman P.L., Rabe C.S., Grant K.A., Valverius P., Hudspith M., Tabakoff B. (1990). Ethanol and the NMDA receptor. Alcohol.

[28] Chandrasekar R. (2013). Alcohol and NMDA receptor: Current research and future direction. Front. Mol. Neurosci..

[29] Bloodgood B.L., Sabatini B.L., van Dongen A.M. (2009). NMDA Receptor-Mediated Calcium Transients in Dendritic Spines. Biology of the NMDA Receptor.

[30] Lovinger D.M., White G., Weight F.F. (1989). Ethanol inhibits NMDA-activated ion current in hippocampal neurons. Science.

[31] Maldve R.E., Zhang T.A., Ferrani-Kile K., Schreiber S.S., Lippmann M.J., Snyder G.L., Fienberg A.A., Leslie S.W., Gonzales R.A., Morrisett R.A. (2002). DARPP-32 and regulation of the ethanol sensitivity of NMDA receptors in the nucleus accumbens. Nat. Neurosci..

[32] Burgos C.F., Muñoz B., Guzman L., Aguayo L.G. (2015). Ethanol effects on glycinergic transmission: From molecular pharmacology to behavior responses. Pharmacol. Res..

[33] Aguayo L.G., van Zundert B., Tapia J.C., Carrasco M.A., Alvarez F.J. (2004). Changes on the properties of glycine receptors during neuronal development. Brain Res. Rev..

[34] Sebe J.Y., Eggers E.D., Berger A.J. (2003). Differential effects of ethanol on GABA(A) and glycine receptor-mediated synaptic currents in brain stem motoneurons. J. Neurophysiol..

[35] Molander A., Löf E., Stomberg R., Ericson M., Söderpalm B. (2005). Involvement of accumbal glycine receptors in the regulation of voluntary ethanol intake in the rat. Alcohol. Clin. Exp. Res..

[36] Spanagel R. (2009). Alcoholism: A Systems Approach From Molecular Physiology to Addictive Behavior. Physiol. Rev..

[37] Perkins D.I., Trudell J.R., Crawford D.K., Alkana R.L., Davies D.L. (2010). Molecular targets and mechanisms for ethanol action in glycine receptors. Pharmacol. Ther..

[38] Buckwalter M.S., Cook S.A., Davisson M.T., White W.F., Camper S.A. (1994). A frameshift mutation in the mouse alpha 1 glycine receptor gene (Glra1) results in progressive neurological symptoms and juvenile death. Hum. Mol. Genet..

[39] Koch M., Kling C., Becker C.M. (1996). Increased startle responses in mice carrying mutations of glycine receptor subunit genes. Neuroreport.

[40] Molander A., Söderpalm B. (2005). Accumbal strychnine-sensitive glycine receptors: An access point for ethanol to the brain reward system. Alcohol. Clin. Exp. Res..

[41] Molander A., Lidö H.H., Löf E., Ericson M., Söderpalm B. (2007). The glycine reuptake inhibitor Org 25935 decreases ethanol intake and preference in male wistar rats. Alcohol Alcohol..

[42] Li J., Nie H., Bian W., Dave V., Janak P.H., Ye J.H. (2012). Microinjection of glycine into the ventral tegmental area selectively decreases ethanol consumption. J. Pharmacol. Exp. Ther..

[43] Rosenbloom M.J., Pfefferbaum A., Sullivan E.V. (1995). Structural Brain Alterations Associated with Alcoholism. Alcohol Health Res. World.

[44] Oscar-Berman M., Marinković K. (2007). Alcohol: Effects on neurobehavioral functions and the brain. Neuropsychol. Rev..

[45] Ditraglia G.M., Press D.S., Butters N., Jernigan T.L., Cermak L.S., Velin R.A., Shear P.K., Irwin M., Schuckit M. (1991). Assessment of olfactory deficits in detoxified alcoholics. Alcohol.

[46] Harper C., Matsumoto I. (2005). Ethanol and brain damage. Curr. Opin. Pharmacol..

[47] Gansler D.A., Harris G.J., Oscar-Berman M., Streeter C., Lewis R.F., Ahmed I., Achong D. (2000). Hypoperfusion of inferior frontal brain regions in abstinent alcoholics: A pilot SPECT study. J. Stud. Alcohol.

[48] Wang G.J., Volkow N.D., Roque C.T., Cestaro V.L., Hitzemann R.J., Cantos E.L., Levy A.V., Dhawan A.P. (1993). Functional importance of ventricular enlargement and cortical atrophy in healthy subjects and alcoholics as assessed with PET, MR imaging, and neuropsychologic testing. Radiology.

[49] Verma R., Kumar C. (2020). Wernicke’s Encephalopathy: Typical Disease with an Atypical Clinicoradiological Manifestation. J. Neurosci. Rural Pract..

[50] Patel A., Biso G.M.N.R., Fowler J.B. (2022). Neuroanatomy, Temporal Lobe.

[51] Moselhy H.F., Georgiou G., Kahn A. (2001). Frontal Lobe Changes in Alcoholism: A Review of the Literature. Alcohol Alcohol..

[52] Sullivan E.V., Marsh L., Mathalon D.H., Lim K.O., Pfefferbaum A. (1996). Relationship between Alcohol Withdrawal Seizures and Temporal Lobe White Matter Volume Deficits. Alcohol. Clin. Exp. Res..

[53] Cardenas V.A., Studholme C., Gazdzinski S., Durazzo T.C., Meyerhoff D.J. (2007). Deformation-based morphometry of brain changes in alcohol dependence and abstinence. NeuroImage.

[54] Waszkiewicz N., Galińska-Skok B., Nestsiarovich A., Kułak-Bejda A., Wilczyńska K., Simonienko K., Kwiatkowski M., Konarzewska B. (2018). Neurobiological Effects of Binge Drinking Help in Its Detection and Differential Diagnosis from Alcohol Dependence. Dis. Markers.

[55] Jean-Richard-Dit-Bressel P., Killcross S., McNally G.P. (2018). Behavioral and neurobiological mechanisms of punishment: Implications for psychiatric disorders. Neuropsychopharmacology.

[56] Davis M., Whalen P.J. (2001). The amygdala: Vigilance and emotion. Mol. Psychiatry.

[57] Delaveau P., Salgado-Pineda P., Wicker B., Micallef-Roll J., Blin O. (2005). Effect of levodopa on healthy volunteers’ facial emotion perception: An FMRI study. Clin. Neuropharmacol..

[58] Konkel A., Cohen N.J. (2009). Relational memory and the hippocampus: Representations and methods. Front. Neurosci..

[59] Sullivan E.V., Marsh L., Mathalon D.H., Lim K.O., Pfefferbaum A. (1995). Anterior hippocampal volume deficits in nonamnesic, aging chronic alcoholics. Alcohol. Clin. Exp. Res..

[60] Harding A.J., Wong A., Svoboda M., Kril J.J., Halliday G.M. (1997). Chronic alcohol consumption does not cause hippocampal neuron loss in humans. Hippocampus.

[61] White A.M., Matthews D.B., Best P.J. (2000). Ethanol, memory, and hippocampal function: A review of recent findings. Hippocampus.

[62] Bartels C., Kunert H.J., Stawicki S., Kröner-Herwig B., Ehrenreich H., Krampe H. (2007). Recovery of hippocampus-related functions in chronic alcoholics during monitored long-term abstinence. Alcohol Alcohol..

[63] Sullivan E.V., Pfefferbaum A. (2009). Neuroimaging of the Wernicke-Korsakoff syndrome. Alcohol Alcohol..

[64] Baker K.G., Harding A.J., Halliday G.M., Kril J.J., Harper C.G. (1999). Neuronal loss in functional zones of the cerebellum of chronic alcoholics with and without Wernicke’s encephalopathy. Neuroscience.

[65] Sullivan E.V. (2003). Compromised pontocerebellar and cerebellothalamocortical systems: Speculations on their contributions to cognitive and motor impairment in nonamnesic alcoholism. Alcohol. Clin. Exp. Res..

[66] Desmond J.E., Chen S.H.A., DeRosa E., Pryor M.R., Pfefferbaum A., Sullivan E.V. (2003). Increased frontocerebellar activation in alcoholics during verbal working memory: An fMRI study. Neuroimage.

[67] Jung M.E. (2015). Alcohol Withdrawal and Cerebellar Mitochondria. Cerebellum.

[68] Luo J. (2015). Effects of Ethanol on the Cerebellum: Advances and Prospects. Cerebellum.

[69] Fritz M., Klawonn A.M., Zahr N.M. (2022). Neuroimaging in alcohol use disorder: From mouse to man. J. Neurosci. Res..

[70] Zahr N.M., Mayer D., Rohlfing T., Hasak M.P., Hsu O., Vinco S., Orduna J., Luong R., Sullivan E.V., Pfefferbaum A. (2010). Brain injury and recovery following binge ethanol: Evidence from in vivo magnetic resonance spectroscopy. Biol. Psychiatry.

[71] Zahr N.M., Mayer D., Rohlfing T., Orduna J., Luong R., Sullivan E.V., Pfefferbaum A. (2013). A mechanism of rapidly reversible cerebral ventricular enlargement independent of tissue atrophy. Neuropsychopharmacology.

[72] Zahr N.M., Pfefferbaum A. (2017). Alcohol’s Effects on the Brain: Neuroimaging Results in Humans and Animal Models. Alcohol Res..

[73] Pfefferbaum A., Zahr N.M., Mayer D., Vinco S., Orduna J., Rohlfing T., Sullivan E.V. (2008). Ventricular expansion in wild-type Wistar rats after alcohol exposure by vapor chamber. Alcohol. Clin. Exp. Res..

[74] Zahr N.M., Mayer D., Vinco S., Orduna J., Luong R., Sullivan E.V., Pfefferbaum A. (2009). In Vivo Evidence for Alcohol-Induced Neurochemical Changes in Rat Brain without Protracted Withdrawal, Pronounced Thiamine Deficiency, or Severe Liver Damage. Neuropsychopharmacology.

[75] Zahr N.M., Mayer D., Rohlfing T., Hsu O., Vinco S., Orduna J., Luong R., Bell R.L., Sullivan E.V., Pfefferbaum A. (2014). Rat strain differences in brain structure and neurochemistry in response to binge alcohol. Psychopharmacology.

[76] Zhou F.C., Bledsoe S., Lumeng L., Li T.K. (1991). Immunostained serotonergic fibers are decreased in selected brain regions of alcohol-preferring rats. Alcohol.

[77] Zhou F.C., Zhang J.K., Lumeng L., Li T.K. (1995). Mesolimbic dopamine system in alcohol-preferring rats. Alcohol.

[78] Miguel-Hidalgo J.J. (2005). Lower Packing Density of Glial Fibrillary Acidic Protein–Immunoreactive Astrocytes in the Prelimbic Cortex of Alcohol-I and Alcohol-Drinking Alcohol-Preferring Rats as Compared with Alcohol-Nonpreferring and Wistar Rats. Alcohol. Clin. Exp. Res..

[79] Gozzi A., Agosta F., Massi M., Ciccocioppo R., Bifone A. (2013). Reduced limbic metabolism and fronto-cortical volume in rats vulnerable to alcohol addiction. Neuroimage.

[80] Nixon K., Crews F.T. (2002). Binge ethanol exposure decreases neurogenesis in adult rat hippocampus. J. Neurochem..

[81] Crews F.T., Mdzinarishvili A., Kim D., He J., Nixon K. (2006). Neurogenesis in adolescent brain is potently inhibited by ethanol. Neuroscience.

[82] Anderson M.L., Nokia M.S., Govindaraju K.P., Shors T.J. (2012). Moderate drinking? Alcohol consumption significantly decreases neurogenesis in the adult hippocampus. Neuroscience.

[83] Ehlers C.L., Liu W., Wills D.N., Crews F.T. (2013). Periadolescent ethanol vapor exposure persistently reduces measures of hippocampal neurogenesis that are associated with behavioral outcomes in adulthood. Neuroscience.

[84] Gass J.T., Glen W.B., McGonigal J.T., Trantham-Davidson H., Lopez M.F., Randall P.K., Yaxley R., Floresco S.B., Chandler L.J. (2014). Adolescent alcohol exposure reduces behavioral flexibility, promotes disinhibition, and increases resistance to extinction of ethanol self-administration in adulthood. Neuropsychopharmacology.

[85] Hansson A.C., Nixon K., Rimondini R., Damadzic R., Sommer W.H., Eskay R., Crews F.T., Heilig M. (2010). Long-term suppression of forebrain neurogenesis and loss of neuronal progenitor cells following prolonged alcohol dependence in rats. Int. J. Neuropsychopharmacol..

[86] Coleman L.G., He J., Lee J., Styner M., Crews F.T. (2011). Adolescent binge drinking alters adult brain neurotransmitter gene expression, behavior, brain regional volumes, and neurochemistry in mice. Alcohol. Clin. Exp. Res..

[87] Kuloglu M., Atmaca M., Tezcan E., Ustundag B., Bulut S. (2002). Antioxidant enzyme and malondialdehyde levels in patients with panic disorder. Neuropsychobiology.

[88] Hovatta I., Tennant R.S., Helton R., Marr R.A., Singer O., Redwine J.M., Ellison J.A., Schadt E.E., Verma I.M., Lockhart D.J. (2005). Glyoxalase 1 and glutathione reductase 1 regulate anxiety in mice. Nature.

[89] Krömer S.A., Keßler M.S., Milfay D., Birg I.N., Bunck M., Czibere L., Panhuysen M., Pütz B., Deussing J.M., Holsboer F. (2005). Identification of Glyoxalase-I as a Protein Marker in a Mouse Model of Extremes in Trait Anxiety. J. Neurosci..

[90] Ditzen C., Jastorff A.M., Kessler M.S., Bunck M., Teplytska L., Erhardt A., Krömer S.A., Varadarajuku J., Targosz B.-S., Sayan-Ayata E.F. (2006). Protein biomarkers in a mouse model extremes in trait anxiety. Mol. Cell. Proteom..

[91] Rammal H., Bouayed J., Younos C., Soulimani R. (2008). The impact of high anxiety level on the oxidative status of mouse peripheral blood lymphocytes, granulocytes and monocytes. Eur. J. Pharmacol..

[92] Rammal H., Bouayed J., Younos C., Soulimani R. (2008). Evidence that oxidative stress is linked to anxiety-related behaviour in mice. Brain Behav. Immun..

[93] Zhang C., Yu Y., Ruan L., Wang C., Pan J., Klabnik J., Lueptow L., Zhang H.-T., O’Donnell J.M., Xu Y. (2015). The roles of phosphodiesterase 2 in the central nervous and peripheral systems. Curr. Pharm. Des..

[94] Urushitani M., Inoue R., Nakamizo T., Sawada H., Shibasaki H., Shimohama S. (2000). Neuroprotective effect of cyclic GMP against radical-induced toxicity in cultured spinal motor neurons. J. Neurosci. Res..

[95] Masood A., Nadeem A., Mustafa S.J., O’Donnell J.M. (2008). Reversal of oxidative stress-induced anxiety by inhibition of phosphodiesterase-2 in mice. J. Pharmacol. Exp. Ther..

[96] Tagliari B., Dos Santos T.M., Cunha A.A., Lima D.D., Delwing D., Sitta A., Vargas C.R., Dalmaz C., Wyse A.T.S. (2010). Chronic variable stress induces oxidative stress and decreases butyrylcholinesterase activity in blood of rats. J. Neural Transm..

[97] Moretti M., Colla A., de Oliveira Balen G., dos Santos D.B., Budni J., de Freitas A.E., Farina M., Rodrigues A.L.S. (2012). Ascorbic acid treatment, similarly to fluoxetine, reverses depressive-like behavior and brain oxidative damage induced by chronic unpredictable stress. J. Psychiatr. Res..

[98] Amr M., El-Mogy A., Shams T., Vieira K., Lakhan S.E. (2013). Efficacy of vitamin C as an adjunct to fluoxetine therapy in pediatric major depressive disorder: A randomized, double-blind, placebo-controlled pilot study. Nutr. J..

[99] McHugh R.K., Weiss R.D. (2019). Alcohol Use Disorder and Depressive Disorders. Alcohol Res..

[100] The Alcohol-Depression Connection: Symptoms, Treatment & More. https://www.healthline.com/health/mental-health/alcohol-and-depression.

[101] Bajpai A., Verma A.K., Srivastava M., Srivastava R. (2014). Oxidative stress and major depression. J. Clin. Diagn. Res..

[102] Salim S. (2017). Oxidative Stress and the Central Nervous System. J. Pharmacol Exp. Ther..

[103] Das S.K., Vasudevan D.M. (2007). Alcohol-induced oxidative stress. Life Sci..

[104] Hulbert A.J., Pamplona R., Buffenstein R., Buttemer W.A. (2007). Life and death: Metabolic rate, membrane composition, and life span of animals. Physiol. Rev..

[105] Ayala A., Muñoz M.F., Argüelles S. (2014). Lipid peroxidation: Production, metabolism, and signaling mechanisms of malondialdehyde and 4-hydroxy-2-nonenal. Oxid. Med. Cell. Longev..

[106] Salim S. (2014). Oxidative stress and psychological disorders. Curr. Neuropharmacol..

[107] Finn D.A., Crabbe J.C. (1997). Exploring Alcohol Withdrawal Syndrome. Alcohol Health Res. World.

[108] Schuckit M.A. (2014). Recognition and management of withdrawal delirium (delirium tremens). N. Engl. J. Med..

[109] Alcohol Withdrawal Syndrome: Causes, Symptoms, and Diagnosis. https://www.healthline.com/health/alcoholism/withdrawal#symptoms.

[110] Rahman A., Paul M. (2022). Delirium Tremens.

[111] Grand Mal Seizure—Symptoms and Causes. Mayo Clinic. https://www.mayoclinic.org/diseases-conditions/grand-mal-seizure/symptoms-causes/syc-20363458.

[112] Treatment of Alcohol Withdrawal Syndrome. https://www.uspharmacist.com/article/treatment-of-alcohol-withdrawal-syndrome.

[113] (2016). How Is Body Temperature Regulated and What Is Fever?. https://www.ncbi.nlm.nih.gov/books/NBK279457/.

[114] Withdrawal Syndromes: Practice Essentials, Background, Pathophysiology. https://emedicine.medscape.com/article/819502-overview#a6.

[115] Tolerance, Dependence, Addiction: What’s the Difference? NIDA Archives. https://archives.drugabuse.gov/blog/post/tolerance-dependence-addiction-whats-difference.

[116] Banerjee N. (2014). Neurotransmitters in alcoholism: A review of neurobiological and genetic studies. Indian J. Hum. Genet..

[117] Gianoulakis C. (2001). Influence of the endogenous opioid system on high alcohol consumption and genetic predisposition to alcoholism. J. Psychiatry Neurosci..

[118] Sprouse-Blum A.S., Smith G., Sugai D., Parsa F.D. (2010). Understanding endorphins and their importance in pain management. Hawaii Med. J..

[119] Dupont A., Cusan L., Garon M., Labrie F., Li C.H. (1977). Beta-endorphin: Stimulation of growth hormone release in vivo. Proc. Natl. Acad. Sci. USA.

[120] Pathan H., Williams J. (2012). Basic opioid pharmacology: An update. Br. J. Pain.

[121] Thiagarajan A.B., Mefford I.N., Eskay R.L. (1989). Single-dose ethanol administration activates the hypothalamic-pituitary-adrenal axis: Exploration of the mechanism of action. Neuroendocrinology.

[122] De Waele J.P., Papachristou D.N., Gianoulakis C. (1992). The alcohol-preferring C57BL/6 mice present an enhanced sensitivity of the hypothalamic beta-endorphin system to ethanol than the alcohol-avoiding DBA/2 mice. J. Pharmacol. Exp. Ther..

[123] De Waele J.P., Gianoulakis C. (1993). Effects of single and repeated exposures to ethanol on hypothalamic beta-endorphin and CRH release by the C57BL/6 and DBA/2 strains of mice. Neuroendocrinology.

[124] Gianoulakis C., Hutchison W.D., Kalant H. (1988). Effects of ethanol treatment and withdrawal on biosynthesis and processing of proopiomelanocortin by the rat neurointermediate lobe. Endocrinology.

[125] Seizinger B.R., Höllt V., Herz A. (1984). Effects of chronic ethanol treatment on the in vitro biosynthesis of pro-opiomelanocortin and its posttranslational processing to beta-endorphin in the intermediate lobe of the rat pituitary. J. Neurochem..

[126] Scanlon M.N., Lazar-Wesley E., Grant K.A., Kunos G. (1992). Proopiomelanocortin messenger RNA is decreased in the mediobasal hypothalamus of rats made dependent on ethanol. Alcohol. Clin. Exp. Res..

[127] Angelogianni P., Gianoulakis C. (1993). Chronic ethanol increases proopiomelanocortin gene expression in the rat hypothalamus. Neuroendocrinology.

[128] Nylander I., Hyytiä P., Forsander O., Terenius L. (1994). Differences between alcohol-preferring (AA) and alcohol-avoiding (ANA) rats in the prodynorphin and proenkephalin systems. Alcohol. Clin. Exp. Res..

[129] Marinelli P.W., Kiianmaa K., Gianoulakis C. (2000). Opioid propeptide mRNA content and receptor density in the brains of AA and ANA rats. Life Sci..

[130] Ng G.Y., O’Dowd B.F., George S.R. (1996). Genotypic differences in mesolimbic enkephalin gene expression in DBA/2J and C57BL/6J inbred mice. Eur. J. Pharmacol..

[131] Jamensky N.T., Gianoulakis C. (1997). Content of Dynorphins and k-Opioid Receptors in Distinct Brain Regions of C57BL/6 and DBA/2 Mice. Alcohol. Clin. Exp. Res..

[132] Fadda P., Tronci S., Colombo G., Fratta W. (1999). Differences in the opioid system in selected brain regions of alcohol-preferring and alcohol-nonpreferring rats. Alcohol. Clin. Exp. Res..

[133] Turchan J., Przewłocka B., Toth G., Lasoń W., Borsodi A., Przewłocki R. (1999). The effect of repeated administration of morphine, cocaine and ethanol on mu and delta opioid receptor density in the nucleus accumbens and striatum of the rat. Neuroscience.

[134] Heyser C.J., Roberts A.J., Schulteis G., Koob G.F. (1999). Central administration of an opiate antagonist decreases oral ethanol self-administration in rats. Alcohol. Clin. Exp. Res..

[135] Anton R.F. (2008). Naltrexone for the management of alcohol dependence. N. Engl. J. Med..

[136] Naltrexone for Alcoholism Treatment. Addiction Center. https://www.addictioncenter.com/alcohol/naltrexone-for-alcoholism-treatment/.

[137] Modesto-Lowe V., Huard J., Conrad C. (2005). Alcohol withdrawal kindling: Is there a role for anticonvulsants?. Psychiatry.

[138] Becker H.C., Diaz-Granados J.L., Weathersby R.T. (1997). Repeated ethanol withdrawal experience increases the severity and duration of subsequent withdrawal seizures in mice. Alcohol.

[139] Verleye M., Heulard I., Gillardin J.M. (2009). The anxiolytic etifoxine protects against convulsant and anxiogenic aspects of the alcohol withdrawal syndrome in mice. Alcohol.

[140] Myrick H., Malcolm R., Randall P.K., Boyle E., Anton R.F., Becker H.C., Randall C.L. (2009). A double-blind trial of gabapentin versus lorazepam in the treatment of alcohol withdrawal. Alcohol. Clin. Exp. Res..

[141] Bourin M. (2020). Mechanism of Action of Valproic Acid and Its Derivatives. https://symbiosisonlinepublishing.com/pharmacy-pharmaceuticalsciences/pharmacy-pharmaceuticalsciences99.pdf.

[142] Becker H.C., Veatch L.M. (2002). Effects of lorazepam treatment for multiple ethanol withdrawals in mice. Alcohol. Clin. Exp. Res..

[143] Veatch L.M., Gonzalez L.P. (1996). Repeated ethanol withdrawal produces site-dependent increases in EEG spiking. Alcohol. Clin. Exp. Res..

[144] Mhatre M.C., McKenzie S.E., Gonzalez L.P. (2001). Diazepam during prior ethanol withdrawals does not alter seizure susceptibility during a subsequent withdrawal. Pharmacol. Biochem. Behav..

[145] Barrons R., Roberts N. (2010). The role of carbamazepine and oxcarbazepine in alcohol withdrawal syndrome. J. Clin. Pharm. Ther..

[146] Slominski A. (2009). On the role of the corticotropin-releasing hormone signalling system in the aetiology of inflammatory skin disorders. Br. J. Dermveatchatol..

[147] Heilig M., Koob G.F. (2007). A key role for corticotropin-releasing factor in alcohol dependence. Trends Neurosci..

[148] Nie Z., Schweitzer P., Roberts A.J., Madamba S.G., Moore S.D., Siggins G.R. (2004). Ethanol Augments GABAergic Transmission in the Central Amygdala via CRF1 Receptors. Science.

[149] Hawley R.J., Nemeroff C.B., Bissette G., Guidotti A., Rawlings R., Linnoila M. (1994). Neurochemical correlates of sympathetic activation during severe alcohol withdrawal. Alcohol. Clin. Exp. Res..

[150] Rasmussen D.D., Wilkinson C.W., Raskind M.A. (2006). Chronic daily ethanol and withdrawal: 6. Effects on rat sympathoadrenal activity during “abstinence”. Alcohol.

[151] Baldwin H.A., Rassnick S., Rivier J., Koob G.F., Britton K.T. (1991). CRF antagonist reverses the “anxiogenic” response to ethanol withdrawal in the rat. Psychopharmacology.

[152] Funk C.K., O’Dell L.E., Crawford E.F., Koob G.F. (2006). Corticotropin-releasing factor within the central nucleus of the amygdala mediates enhanced ethanol self-administration in withdrawn, ethanol-dependent rats. J. Neurosci..

[153] Funk C.K., Zorrilla E.P., Lee M.J., Rice K.C., Koob G.F. (2007). Corticotropin-Releasing Factor 1 Antagonists Selectively Reduce Ethanol Self-Administration in Ethanol-Dependent Rats. Biol. Psychiatry.

[154] Overstreet D.H., Knapp D.J., Breese G.R. (2004). Modulation of multiple ethanol withdrawal-induced anxiety-like behavior by CRF and CRF1 receptors. Pharmacol. Biochem. Behav..

[155] Spina M.G., Merlo-Pich E., Akwa Y., Balducci C., Basso A., Zorrilla E., Britton K., Rivier L., Vale W., Koob G. (2002). Time-dependent induction of anxiogenic-like effects after central infusion of urocortin or corticotropin-releasing factor in the rat. Psychopharmacology.

[156] Lê A.D., Harding S., Juzytsch W., Watchus J., Shalev U., Shaham Y. (2000). The role of corticotrophin-releasing factor in stress-induced relapse to alcohol-seeking behavior in rats. Psychopharmacology.

[157] Liu X., Weiss F. (2002). Additive effect of stress and drug cues on reinstatement of ethanol seeking: Exacerbation by history of dependence and role of concurrent activation of corticotropin-releasing factor and opioid mechanisms. J. Neurosci..

[158] Lê A.D., Harding S., Juzytsch W., Fletcher P.J., Shaham Y. (2002). The role of corticotropin-releasing factor in the median raphe nucleus in relapse to alcohol. J. Neurosci..

[159] Yu H., Chen Z. (2011). The role of BDNF in depression on the basis of its location in the neural circuitry. Acta Pharmacol. Sin..

[160] Park H., Poo M.M. (2013). Neurotrophin regulation of neural circuit development and function. Nat. Rev. Neurosci..

[161] Dwivedi Y. (2009). Brain-derived neurotrophic factor: Role in depression and suicide. Neuropsychiatr. Dis. Treat..

[162] Roni M.A., Rahman S. (2017). Lobeline attenuates ethanol abstinence-induced depression-like behavior in mice. Alcohol.

[163] Ghitza U.E., Zhai H., Wu P., Airavaara M., Shaham Y., Lu L. (2010). Role of BDNF and GDNF in drug reward and relapse: A review. Neurosci. Biobehav. Rev..

[164] Hou L., Guo Y., Lian B., Wang Y., Li C., Wang G., Li Q., Pang J., Sun H., Sun L. (2018). Synaptic Ultrastructure Might Be Involved in HCN^1^-Related BDNF mRNA in Withdrawal-Anxiety after Ethanol Dependence. Front. Psychiatry.

[165] Liu F.G., Hu W.F., Wang J.L., Wang P., Gong Y., Tong L.J., Jiang B., Zhang W., Qin Y.-B., Chen Z. (2017). Z-Guggulsterone Produces Antidepressant-Like Effects in Mice through Activation of the BDNF Signaling Pathway. Int. J. Neuropsychopharmacol..

[166] Chen Z.Y., Patel P.D., Sant G., Meng C.-X., Teng K.K., Hempstead B.L., Lee F.S. (2004). Variant brain-derived neurotrophic factor (BDNF) (Met66) alters the intracellular trafficking and activity-dependent secretion of wild-type BDNF in neurosecretory cells and cortical neurons. J. Neurosci..

[167] Monteggia L.M., Luikart B., Barrot M., Theobold D., Malkovska I., Nef S., Parada L.F., Nestler E.J. (2007). Brain-Derived Neurotrophic Factor Conditional Knockouts Show Gender Differences. Biol. Psychiatry.

[168] Duman R.S., Monteggia L.M. (2006). A Neurotrophic Model for Stress-Related Mood Disorders. Biol. Psychiatry.

[169] Videbech P., Ravnkilde B. (2004). Hippocampal volume and depression: A meta-analysis of MRI studies. Am. J. Psychiatry.

[170] Lucassen P.J., Müller M.B., Holsboer F., Bauer J., Holtrop A., Wouda J., Hoogendijk W.J.G., De Kloet E.R., Swaab D.F. (2001). Hippocampal apoptosis in major depression is a minor event and absent from subareas at risk for glucocorticoid overexposure. Am. J. Pathol..

[171] Stockmeier C.A., Mahajan G.J., Konick L.C., Overholser J.C., Jurjus G.J., Meltzer H.Y., Uylings H.B.M., Friedman L., Rajkowska G. (2004). Cellular changes in the postmortem hippocampus in major depression. Biol. Psychiatry.

[172] MacQueen G.M., Yucel K., Taylor V.H., Macdonald K., Joffe R. (2008). Posterior hippocampal volumes are associated with remission rates in patients with major depressive disorder. Biol. Psychiatry.

[173] Kronmüller K.T., Pantel J., Köhler S., Victor D., Giesel F., Magnotta V.A., Mundt C., Essig M., Schröder J. (2008). Hippocampal volume and 2-year outcome in depression. Br. J. Psychiatry.

[174] Dwivedi Y., Rao J.S., Rizavi H.S., Kotowski J., Conley R.R., Roberts R.C., Tamminga C.A., Pandey G.N. (2003). Abnormal expression and functional characteristics of cyclic adenosine monophosphate response element binding protein in postmortem brain of suicide subjects. Arch. Gen. Psychiatry.

[175] Chen B., Dowlatshahi D., MacQueen G.M., Wang J.-F., Young L.T. (2001). Increased hippocampal bdnf immunoreactivity in subjects treated with antidepressant medication. Biol. Psychiatry.

[176] Russo-Neustadt A.A., Alejandre H., Garcia C., Ivy A.S., Chen M.J. (2004). Hippocampal brain-derived neurotrophic factor expression following treatment with reboxetine, citalopram, and physical exercise. Neuropsychopharmacology.

[177] Nibuya M., Morinobu S., Duman R.S. (1995). Regulation of BDNF and trkB mRNA in rat brain by chronic electroconvulsive seizure and antidepressant drug treatments. J. Neurosci..

[178] Shirayama Y., Chen A.C., Nakagawa S., Russell D.S., Duman R.S. (2002). Brain-derived neurotrophic factor produces antidepressant effects in behavioral models of depression. J. Neurosci..

[179] Deltheil T., Guiard B.P., Cerdan J., David D.J., Tanaka K.F., Repérant C., Guilloux J.-P., Coudoré F., Hen R., Gardier A.M. (2008). Behavioral and serotonergic consequences of decreasing or increasing hippocampus brain-derived neurotrophic factor protein levels in mice. Neuropharmacology.

[180] Schmidt H.D., Duman R.S. (2010). Peripheral BDNF produces antidepressant-like effects in cellular and behavioral models. Neuropsychopharmacology.

[181] Poduslo J.F., Curran G.L. (1996). Permeability at the blood-brain and blood-nerve barriers of the neurotrophic factors: NGF, CNTF, NT-3, BDNF. Brain Res. Mol. Brain Res..

[182] Zhang Y., Pardridge W.M. (2001). Conjugation of brain-derived neurotrophic factor to a blood-brain barrier drug targeting system enables neuroprotection in regional brain ischemia following intravenous injection of the neurotrophin. Brain Res..

[183] Pan W., Banks W.A., Fasold M.B., Bluth J., Kastin A.J. (1998). Transport of brain-derived neurotrophic factor across the blood-brain barrier. Neuropharmacology.

[184] Pearse R.N., Swendeman S.L., Li Y., Rafii D., Hempstead B.L. (2005). A neurotrophin axis in myeloma: TrkB and BDNF promote tumor-cell survival. Blood.

[185] Eisch A.J., Bolaños C.A., de Wit J., Simonak R.D., Pudiak C.M., Barrot M., Verhaagen J., Nestler E.J. (2003). Brain-derived neurotrophic factor in the ventral midbrain-nucleus accumbens pathway: A role in depression. Biol. Psychiatry.

[186] Siuciak J.A., Lewis D.R., Wiegand S.J., Lindsay R.M. (1997). Antidepressant-like effect of brain-derived neurotrophic factor (BDNF). Pharmacol. Biochem. Behav..

[187] Hoshaw B.A., Malberg J.E., Lucki I. (2005). Central administration of IGF-I and BDNF leads to long-lasting antidepressant-like effects. Brain Res..

[188] Hacioglu G., Senturk A., Ince I., Alver A. (2016). Assessment of oxidative stress parameters of brain-derived neurotrophic factor heterozygous mice in acute stress model. Iran. J. Basic Med. Sci..

[189] Parthasarathy R., Kattimani S., Sridhar M.G. (2015). Oxidative stress during alcohol withdrawal and its relationship with withdrawal severity. Indian J. Psychol. Med..

[190] Gonzaga N.A., Mecawi A.S., Antunes-Rodrigues J., de Martinis B.S., Padovan C.M., Tirapelli C.R. (2015). Ethanol withdrawal increases oxidative stress and reduces nitric oxide bioavailability in the vasculature of rats. Alcohol.

[191] Assis V.O., Gonzaga N.A., Silva C.B.P., Pereira L.C., Padovan C.M., Tirapelli C.R. (2020). Ethanol Withdrawal Alters the Oxidative State of the Heart Through AT1-Dependent Mechanisms. Alcohol Alcohol..

[192] Iovieno N., Tedeschini E., Bentley K.H., Evins A.E., Papakostas G.I. (2011). Antidepressants for major depressive disorder and dysthymic disorder in patients with comorbid alcohol use disorders: A meta-analysis of placebo-controlled randomized trials. J. Clin. Psychiatry.

[193] Nunes E.V., Levin F.R. (2004). Treatment of depression in patients with alcohol or other drug dependence: A meta-analysis. JAMA.

[194] Agabio R., Trogu E., Pani P.P. (2018). Antidepressants for the treatment of people with co-occurring depression and alcohol dependence. Cochrane Database Syst. Rev..

[195] Torrens M., Fonseca F., Mateu G., Farré M. (2005). Efficacy of antidepressants in substance use disorders with and without comorbid depression. A systematic review and meta-analysis. Drug Alcohol Depend..

[196] Nunes E.V., Quitkin F.M., Donovan S.J., Deliyannides D., Ocepek-Welikson K., Koenig T., Brady R., McGrath P.J., Woody G. (1998). Imipramine treatment of opiate-dependent patients with depressive disorders. A placebo-controlled trial. Arch. Gen. Psychiatry.

[197] Petrakis I., Ralevski E., Nich C., Levinson C., Carroll K., Poling J., Rounsaville B., VA VISN I MIRECC Study Group (2007). Naltrexone and disulfiram in patients with alcohol dependence and current depression. J. Clin. Psychopharmacol..

[198] Lejoyeux M., Lehert P. (2011). Alcohol-use disorders and depression: Results from individual patient data meta-analysis of the acamprosate-controlled studies. Alcohol Alcohol..

[199] Pettinati H.M., Oslin D.W., Kampman K.M., Dundon W.D., Xie H., Gallis T.L., Dackis C.A., O’Brien C.P. (2010). A double-blind, placebo-controlled trial combining sertraline and naltrexone for treating co-occurring depression and alcohol dependence. Am. J. Psychiatry.

[200] Witte J., Bentley K., Evins A.E., Clain A.J., Baer L., Pedrelli P., Fava M., Mischoulon D. (2012). A Randomized, Controlled, Pilot Study of Acamprosate Added to Escitalopram in Adults with Major Depressive Disorder and Alcohol Use Disorder. J. Clin. Psychopharmacol..

